# A New PqsR Inverse Agonist Potentiates Tobramycin Efficacy to Eradicate *Pseudomonas aeruginosa* Biofilms

**DOI:** 10.1002/advs.202004369

**Published:** 2021-03-18

**Authors:** Christian Schütz, Duy‐Khiet Ho, Mostafa Mohamed Hamed, Ahmed Saad Abdelsamie, Teresa Röhrig, Christian Herr, Andreas Martin Kany, Katharina Rox, Stefan Schmelz, Lorenz Siebenbürger, Marius Wirth, Carsten Börger, Samir Yahiaoui, Robert Bals, Andrea Scrima, Wulf Blankenfeldt, Justus Constantin Horstmann, Rebekka Christmann, Xabier Murgia, Marcus Koch, Aylin Berwanger, Brigitta Loretz, Anna Katharina Herta Hirsch, Rolf Wolfgang Hartmann, Claus‐Michael Lehr, Martin Empting

**Affiliations:** ^1^ Helmholtz‐Institute for Pharmaceutical Research Saarland (HIPS)‐Helmholtz Centre for Infection Research (HZI) Campus E8.1 Saarbrücken 66123 Germany; ^2^ German Centre for Infection Research (DZIF) Partner Site Hannover‐Braunschweig Saarbrücken 66123 Germany; ^3^ Department of Pharmacy Saarland University Campus E8.1 Saarbrücken 66123 Germany; ^4^ Chemistry of Natural and Microbial Products Department National Research Centre Dokki Cairo 12622 Egypt; ^5^ Department of Internal Medicine V – Pulmonology Allergology and Critical Care Medicine Saarland University Building 41 Homburg 66421 Germany; ^6^ Department of Chemical Biology (CBIO) Helmholtz Centre for Infection Research (HZI) Inhoffenstr. 7 Braunschweig 38124 Germany; ^7^ Department of Structure and Function of Proteins (SFPR) Helmholtz Centre for Infection Research (HZI) Inhoffenstr. 7 Braunschweig 38124 Germany; ^8^ PharmBioTec GmbH Science Park 1 Saarbrücken 66123 Germany; ^9^ Leibniz Institute for New Materials Campus D2.2 Saarbrücken 66123 Germany; ^10^ Department of Bioengineering School of Medicine University of Washington Seattle WA 98195 USA; ^11^ Gaiker Technology Centre Zamudio Bizkaia 48170 Spain

**Keywords:** biofilm inhibition, nanoparticles, *Pseudomonas aeruginosa*, quorum sensing

## Abstract

*Pseudomonas aeruginosa* (PA) infections can be notoriously difficult to treat and are often accompanied by the development of antimicrobial resistance (AMR). Quorum sensing inhibitors (QSI) acting on PqsR (MvfR) – a crucial transcriptional regulator serving major functions in PA virulence – can enhance antibiotic efficacy and eventually prevent the AMR. An integrated drug discovery campaign including design, medicinal chemistry‐driven hit‐to‐lead optimization and in‐depth biological profiling of a new QSI generation is reported. The QSI possess excellent activity in inhibiting pyocyanin production and PqsR reporter‐gene with IC_50_ values as low as 200 and 11 × 10^−9^ m, respectively. Drug metabolism and pharmacokinetics (DMPK) as well as safety pharmacology studies especially highlight the promising translational properties of the lead QSI for pulmonary applications. Moreover, target engagement of the lead QSI is shown in a PA mucoid lung infection mouse model. Beyond that, a significant synergistic effect of a QSI‐tobramycin (Tob) combination against PA biofilms using a tailor‐made squalene‐derived nanoparticle (NP) formulation, which enhance the minimum biofilm eradicating concentration (MBEC) of Tob more than 32‐fold is demonstrated. The novel lead QSI and the accompanying NP formulation highlight the potential of adjunctive pathoblocker‐mediated therapy against PA infections opening up avenues for preclinical development.

## Graphical abstract

The discovery and profiling of a novel *Pseudomonas aeruginosa*‐targeting Quorum Sensing Inhibitor (QSI) with high anti‐virulence efficacy, favorable DMPK and no overt safety pharmacology is reported. The in vivo target engagement of the lead QSI in a mucoid lung‐infection mouse model is demonstrated. Importantly, the application of a state‐of‐the‐art squalene‐based nanocarrier formulation boosts tobramycin susceptibility of biofilms.

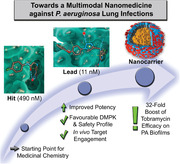

## 1 Introduction

*Pseudomonas aeruginosa* (PA) – a ubiquitous Gram‐negative pathogen – is able to colonize almost any part of the human body and causes severe acute and chronic hospital‐acquired infections. According to a recent meta‐analysis, PA is one of the most frequent pathogens developing pandrug‐resistant strains, which accounts for up to one‐third of all cases worldwide.^[^ ^1^ ^]^ Moreover, PA strains showing susceptibility to anti‐pseudomonal drugs in their planktonic phenotype, however, become notoriously difficult to fully eradicate in clinical settings due to their ability to self‐generate recalcitrant biofilms.^[^ ^2^ ^]^ These persistent and recurring PA infections are frequently associated with chronic lung diseases, like cystic fibrosis (CF), bronchiectasis and chronic obstructive pulmonary disease (COPD).

The paradigmatic case is CF to which chronic PA infections dramatically reduce life expectancy and are a leading cause of death. Furthermore, the first diagnosis of this pathogen leads to a worsened prognosis accompanied by reduced quality of life as well as a high healthcare burden due to increased numbers of hospital stays.^[^ ^2^ ^]^ Unfortunately, currently approved inhaled antibiotics for CF including tobramycin (Tob), aztreonam, colistin, and levofloxacin, often fail to fully eradicate PA from the lungs after primary colonization. Indeed, delay of recurrence rather than eradication is a common therapy endpoint indicating the regular failure of treatment by antibiotics.^[^ ^3^ ^]^ Moreover, the prolonged and repeated courses of inhaled antibiotics, e.g., 28‐day on / 28‐day off cycles of Tob, are not without undesired adverse effects and can promote bacterial resistance. As numbers of PA‐related antimicrobial resistance (AMR) are increasing, the World Health Organization (WHO) categorized carbapenem‐resistant PA as one of the highest priority pathogens for which novel treatment options are urgently needed.^[^ ^4^ ^]^

**Figure 1:**
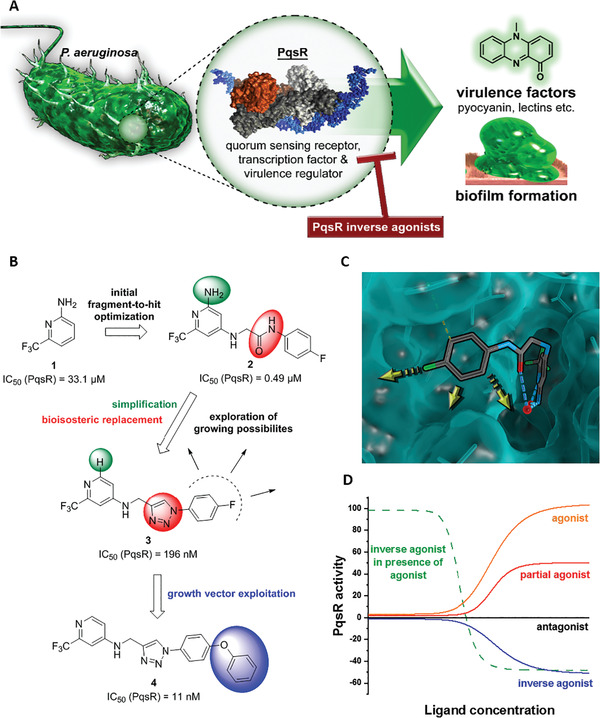
PqsR inverse agonists as pathoblockers and hit‐to‐lead optimization strategy. A) Schematic representation of the mode of action. B) Bioisosteric replacement and structural simplification of hit **2** lead to **3**. The discovery and exploitation of a growth vector‐enabled identification of **4**. C) 3D model of compound **3** in complex with PqsR^91‐319^ derived from related X‐ray structure (PDB entry 6Q7W). D) Pharmacological profile of various PqsR ligands.

In the present study, we set out to provide a basis for improved therapy of chronic PA lung infections. To this end, we employed the so‐called anti‐virulence or pathoblocker approach by targeting a virulence‐controlling regulatory network referred to as quorum sensing (QS).^[^ ^5^ ^]^ Notably, this strategy could lead to less resistance development and reduced side effects, as the pathoblocker synergizes the antibiotic efficacy yet does not affect bacterial viability itself.^[^ ^5^, ^6^ ^]^ QS is a cell‐density‐dependent intercellular communication system making use of diffusible signal molecules, involved in biofilm formation, and also required for full pathogenicity.^[^ ^5^ ^]^ There are four cell‐to‐cell communication systems present in PA, which are referred to as *las*, *rhl*, *pqs*, and *iqs*. These systems are all intertwined, and especially the *las*, *rhl*, and *pqs* QS systems have been the subject of studies exploring these as therapeutic targets.^[^ ^5^, ^7^ ^]^ The latter is rather unique and only found in *Pseudomonas* as well as *Burkholderia spp*., which makes use of alkylquinolone‐based metabolites as signaling molecules. The eponymous PQS (*Pseudomonas* quinolone signal, 2‐heptyl‐3‐hydroxy‐4(1*H*)‐quinolone) and its biosynthetic precursor HHQ (2‐heptyl‐4‐quinolone) are both QS auto‐inducers acting as agonists on the transcriptional regulator PqsR (also referred to as MvfR, **Figure** **1**A).^[^ ^7^, ^8^ ^]^ Due to its central role in virulence regulation, PqsR has been investigated in various studies as a promising new target for treating PA infections. It has been shown that small molecular agents, which act as inverse agonists on PqsR and thus inhibit QS (Figure 1A), are able to reduce the production of the important virulence factor pyocyanin and the auto‐inducing alkylquinolones.^[^ ^9^ ^]^ Moreover, interference with PQS system can prevent biofilm formation and reduce levels of extracellular DNA (eDNA), which are two important mechanisms in PAs tolerance toward antibiotic treatment.^[^ ^10^ ^]^ As a consequence, QS inhibitors (QSI) can enhance antibiotic efficacy against PA biofilms.^[^ ^11^, ^12^ ^]^ This exciting feature renders them attractive for devising an adjunctive treatment, for example, with inhaled standard‐of‐care (SoC) aminoglycoside antibiotic Tob. Pharmacokinetic‐pharmacodynamic (PK/PD) correlation studies in humans have shown that the ratio of the peak exposure to the minimal inhibitory concentration (MIC) toward the respective bacterial strain is a major predictor for the success of aminoglycoside treatment.^[^ ^13^ ^]^ In other words, once the MIC is lowered, e.g., due to the presence of a potentiating agent, a better outcome of backbone antibiosis is more likely at the same dose/exposure. In the biofilm‐related chronic lung‐infection settings, the planktonic MIC value must however be replaced by the minimum biofilm eradicating concentration (MBEC) which in the case of Tob is much higher than the MIC (up to 1000‐fold depending on testing conditions).^[^ ^14^ ^]^ Hence, decreasing such a high MBEC value of an antibiotic by co‐administering a QSI holds great potential in lowering the risk of subsequent resistance development, and eventually improving the clinical outcome.

We previously introduced a novel class of QSI originating from a fragment‐based screening hit **1** (Figure 1B).^[^ ^15^ ^]^ Initial optimization yielded a small‐molecule, which was very efficient in attenuating PA virulence and displayed a promising starting point for a hit‐to‐lead campaign, ultimately resulting in an improved hit **2** with potential for further medicinal chemistry‐driven optimization (Figure 1B).^[^ ^15^ ^]^ Herein, we report the successful generation of a lead QSI including in‐depth ADMET profiling. We show that the new chemical matter not only provides potent anti‐virulence efficacy but also has favorable characteristics regarding in vitro and in vivo PK that are suitable for the applications in the lungs, while showing no overt findings in in vitro safety pharmacology assays.

Beyond that, we were able to combine the lead QSI in a self‐assembling squalenyl hydrogen sulfate nanoparticle (SqNP) system together with Tob at high loading capacity (LC). Importantly, this new dual‐loaded QSI‐antibiotic NP system was superior to the previously reported Tob and a first‐generation alkylquinolone‐derived QSI composite^[^ ^12^ ^]^ enhancing the Tob MBEC value by at least 32‐fold.

In order to show in vivo efficacy of pathoblockers in general and QSI in particular, the application of predictive models is key.^[^ ^16^ ^]^ The anti‐virulence active agent does not interfere with bacterial viability. Hence, typical study endpoints like bacterial load do not reflect the inherent mode‐of‐action. Especially, murine models of PA lung infection are challenging to establish. Most simulate a rather acute setting with short infection times of only 24 to 48 h, and only a few are able to establish chronic colonization scenarios.^[^ ^17^ ^]^ We herein report the application of a mucoid lung infection model in mice, which has an infection time up to 72 h and is suitable for assessing target engagement of PQS‐targeting QSI. This enabled us to determine QS suppression in the lung compartment by quantifying the levels of the QS‐associated metabolites three days post‐infection.^[^ ^16^ ^]^ These included the main alkylquinolone signal molecules PQS and HHQ (vide supra) as well as a close congener HQNO (2‐heptyl‐4‐quinolinol‐1‐oxide).^[^ ^10^ ^]^ These outcomes are of clinical relevance, as the aforementioned metabolites were suggested to be prognostic biomarkers for PA infections in CF patients.^[^ ^18^ ^]^ By this means, we qualified our frontrunner QSI as an in vivo active pathoblocker.

Taken together, this study marks a cornerstone in the translation of QS inhibitor‐based therapies as it brings together the discovery of a new class of frontrunner QSI, its extensive in vitro and in vivo profiling while finally arriving at a fit‐for‐purpose NP‐based formulation.

## 2 Results and Discussion

### 2.1 Design, Hit‐To‐Lead Optimization and Structure‐Activity Relationship (SAR) of the QSI

The following section describes the steps of medicinal chemistry‐driven optimization starting from the previously identified PqsR inverse agonist **2** (Figure 1B).^[^ ^15^ ^]^ Our efforts arrived at lead QSI **4** (Figure 1B), which was then subject to in‐depth biological profiling in the subsequent sections.

A first consideration regarding the optimization of hit compound **2** involved the replacement of the amide bond, as it is inherently prone to enzymatic hydrolysis and might lead to undesirable aniline metabolites.^[^ ^15^, ^19^ ^]^ Our strategy aimed at installing a bioisosteric replacement followed by a fragment‐growing approach. Triazoles have been described as amide mimics^[^ ^20^ ^]^ and were, hence, selected for our rational design approach. Indeed, we were successful in exploiting a 1,4‐disubstituted 1,2,3‐triazole motif for this purpose, which is easily accessible via copper(I)‐catalyzed alkyne‐azide cycloaddition (CuAAC), making it suitable for a combinatorial chemistry approach. We reduced synthetic complexity by omitting the 2‐amino function in the pyridine head group, yielding compound **3** (Figure 1B).

Using a co‐crystal structure of an alkyl derivative, we modeled the binding mode of **2** (Figure 1C).^[^ ^15^ ^]^ Based on these structural insights, we decided to explore the possibility to further enlarge the compound in the Eastern arene motif (Figure 1B). The synthesized compounds were then evaluated in a heterologous reporter‐gene assay in *E. coli* to investigate their on‐target activity (**Table** **1**). With this assay, we were able to evaluate the pharmacological profile of tested compounds and determine whether they act as agonists, antagonists, inverse agonists, or biphasic modulators. The innate receptor PqsR has a basal activity that can only be abolished by inverse agonists (Figure 1D). It is important not only to antagonize PqsR, but also to revert its activity below the basal level. In this scenario, an effective interruption of downstream processes like pyocyanin production is achieved by inverse agonists.^[^ ^10^ ^]^ To our satisfaction, the initial structural modifications proved to be efficient, leading to a threefold increase in activity of **3** compared to the parent compound **2** (Figure 1B). In the next steps, we investigated the three possible substitution positions with chlorinated derivatives **7**–**9** (Table 1). It became evident that the *para*‐substitution motif was suitable for growing. This was surprising, as this direction seemed to be occluded by residues Ile186 and Leu189 in the X‐ray structure and modeled binding mode (Figure 1C). Nevertheless, we installed a number of substituents having various chemical properties in this 4‐position (shown in derivatives **9**–**15**, Table 1). Indeed, this attempt proved successful, as we discovered phenoxy‐modified compound **4**, which showed a highly improved potency at IC_50_ of 11 × 10^−9^ m. The suitability of this motif has also been described previously.^[^ ^21^, ^22^ ^]^ Changing the oxygen linker to nitrogen (shown in derivative **15**, Table 1) in the opposite decreased the activity. Furthermore, 3,4‐disubstituted patterns were evaluated. Combining the phenoxy motif with a chlorine substituent led to equipotent compound **19**. A more prominent impact of chlorine in 3‐position was observed for shorter *para*‐substituents. Compound **17** showed 10‐fold higher activity than monosubstituted **11**. Notably, this effect did not translate to trifluoromethoxy‐decorated compound **18** whose IC_50_ value was improved only two‐fold compared to its parent compound **12**. It is worth mentioning that we observed non‐additive effects of modifications resulting in a non‐linear SAR.

**Table 1:** Biological evaluation of optimized compounds (quorum sensing inhibitors – QSI). On‐target activity was determined in an *Escherichia coli* (*E. coli*)‐based reporter‐gene assay. Pyocyanin inhibition was determined in a *Pseudomonas aeruginosa*‐based assay. Confidence intervals of 95% are shown in brackets

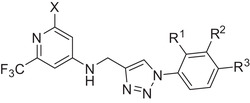						
Compound	X	R^1^	R^2^	R^3^	IC_50_ PqsR	IC_50_ pyocyanin
**3**	H	H	H	F	196 × 10^−9^ m [155–248]	> 5 × 10^−6^ m
**5**	H	H	F	H	inactive	> 5 × 10^−6^ m
**6**	H	F	H	H	inactive	> 10 × 10^−6^ m
**7**	H	Cl	H	H	inactive	> 5 × 10^−6^ m
**8**	H	H	Cl	H	123 × 10^−9^ m [98–153]	> 2.5 × 10^−6^ m
**9**	H	H	H	Cl	79 × 10^−9^ m [64–97]	7.73 × 10^−6^ m [5.65–9.81]
**10**	H	H	H	OH	inactivea)	n.d.^b)^
**11**	H	H	H	OMe	81 × 10^−9^ m [57–104]	2.5 × 10^−6^ m [2.83–3.89]
**12**	H	H	H	OCF_3_	30 × 10^−9^ m [22–38]	1.18 × 10^−6^ m [9.72–1.39]
**13**	H	H	H	CF_3_	129 × 10^−9^ m	> 5 × 10^−6^ m
**14**	H	H	H	CN	655 × 10^−9^ m [383–1120]	> 10 × 10^−6^ m
**4**	H	H	H	OPh	11 × 10^−9^ m [11–12]	199 × 10^−9^ m [146–252]
**15**	H	H	H	NPh	31 × 10^−9^ m	1.06 × 10^−6^ m [0.68–1.44]
**16**	H	H	Cl	Cl	24 × 10^−9^ m [17–33]	354 × 10^−9^ m [259–483]
**17**	H	H	Cl	OMe	8 × 10^−9^ m [7–10]	350 × 10^−9^ m [289–350]
**18**	H	H	Cl	OCF_3_	15 × 10^−9^ m [10–20]	438 × 10^−9^ m [330–547]
**19**	H	H	Cl	OPh	12 × 10^−9^ m [10–15]	181 × 10^−9^ m [152–211]
**20**	NH_2_	H	H	OPh	n.d.^b)^	351 × 10^−9^ m [135–620]

^a)^ inactive = > 10 × 10^−6^ m

^b)^ n.d. = not determined.

In addition to the reporter‐gene assay, we tested the compounds for their ability to reduce pyocyanin levels in PA (Table 1). It is worthwhile mentioning that in contrast to the employed *E. coli* DH5*α*, known to possess a more permeable cell wall than PA, a decrease in activity for this anti‐virulence assay in PA was expected. The fully functional barrier of the Gram‐negative cell wall present in PA14 reduces the intracellular accumulation of test compounds. In accordance with the on‐target activities (reporter‐gene assay), **4** and **19** were almost equipotent. Surprisingly, **17** being the only compound with a single‐digit nanomolar IC_50_ proved to be less active than **4**. The activity of **18** was also drastically decreased in terms of pyocyanin inhibition, even though its on‐target activity was comparable to **4**. We hypothesize that this observation was due to decreased cellular uptake of less lipophilic compounds violating the eNTRy rules,^[^ ^23^ ^]^ as well as efflux reasons. Finally, the amine presence in the head group of hit compound **2** was not crucial for cellular activity and rather proved to be non‐beneficial, inhibiting pyocyanin production at an IC_50_ of 351 × 10^−9^ m (20).

Additional secondary biological activity tests showed that compounds **4** and **17** were able to suppress signal molecule synthesis as well as eDNA secretion in PA (**Table** **2**). In accordance with observed activity on pyocyanin, **4** demonstrated higher efficacy in these cell‐based assays (IC_50_(HHQ) = 0.37 ± 0.07 × 10^−6^ m, IC_50_(PQS) = 1.13 × 10^−6^ m ± 0.07, IC_50_(eDNA) = 0.22 ± 0.05 × 10^−6^ m). Nevertheless, **17** was still reasonably active against these downstream biomarkers (IC_50_(HHQ) = 1.32 ± 0.39 × 10^−6^ m, IC_50_(PQS) = 2.08 ± 0.37 × 10^−6^ m, IC_50_(eDNA) = 1.6 ± 1.1 × 10^−6^ m).

**Table 2:** Biological‐activity, metabolic‐stability and safety‐pharmacology profiling. Dose response curves represent means ± SD of at least 3 independent experiments

	Parameter	4	17
Efficacy	Reporter‐gene assay (on target effect in heterologous system *E. coli*)	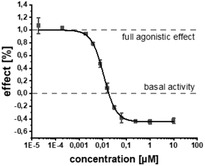	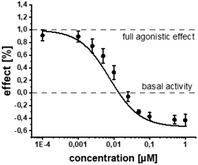
	Pyocyanin inhibition (anti‐virulence effect in PA)	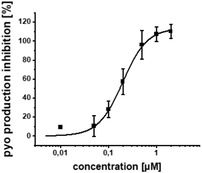	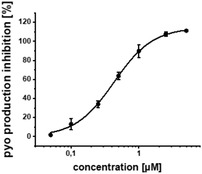
	Inhibition of alkylquinolones PQS and HHQ (suppression of QS metabolites in PA)	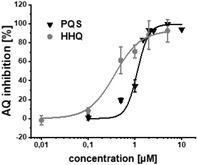	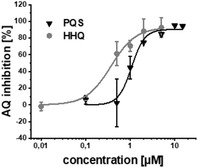
	Inhibition of eDNA production in biofilm (suppression of biofilm component in PA)	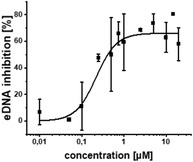	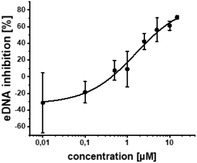
In Vitro DMPK and Safety Pharmacology	Kinetic solubility *S*_kin_	7.7 × 10^−6^ m	64.1 × 10^−6^ m
	Metabolic stability *t*_1/2_ S9 human/mouse	169/29 min	42/11 min
	Permeability P_app_ (using Calu‐3 lung epithelial cells)	5.30 × 10^–6^ cm s^−1^	11.41 × 10^–6^ cm s^−1^
	CYP inhibition 3A4/2D6/1A/2C9/2C19 (important off‐targets for drug metabolism and drug–drug interactions)	15/>25/>25/16.4/20.3 × 10^−6^ m	>25/>25/22.1/>25/3.55 × 10^−6^ m
	hERG inhibition (important human off‐target for cardiotoxicity)	>25 × 10^−6^ m	>25 × 10^−6^ m
	Cytotoxicity (in hepatic cells, HepG2)	55% viable cells @25 × 10^−6^ m	68% viable cells @75 × 10^−6^ m

### 2.2 Co‐Crystal Structure of QSI **4** in Complex with the Transcriptional Regulator PqsR^91‐319^

**Figure 2:**
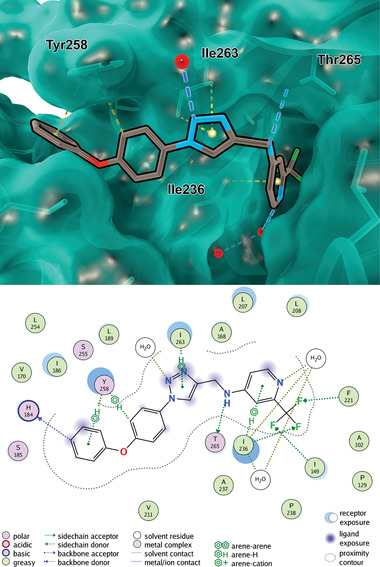
X‐ray crystallography. A) Cocrystal structure of compound **4** in complex with PqsR^91‐319^ (PDB ID: 6YIZ) at a resolution of 2.15 Å and main interactions. B) Interactions of compound **4** with PqsR^91‐319^.

The exploited growth vector was further endorsed by a co‐crystal structure of **4** in complex with the ligand binding domain of the transcriptional regulator PqsR^91‐319^ (**Figure** **2**). Herein, we observed the opening of an additional pocket upon binding. The *para*‐installed phenoxy moiety causes a 4 Å movement of Ile186 as measured by *α*‐carbon distance. Such observations of receptor flexibility and induced fit reshaping of the ligand‐binding site are fascinating and likely the reason for the observed non‐additive SAR.

### 2.3 In Vitro Drug Metabolism and Pharmacokinetics (DMPK) and Safety Pharmacology of the QSI

Despite the challenges posed by the encountered non‐linear SAR, we were successful in achieving high anti‐virulence efficacy in the same ballpark as other reported QSI.^[^ ^21^, ^22^ ^]^ High potency, however, is only one criterion of an active pharmaceutical ingredient. Ideally, lead generation efforts include detailed analysis of ADMET and safety pharmacology features.^[^ ^24^ ^]^ We conducted a cascade of in vitro DMPK and safety pharmacology profiling tests with ascending complexity.

First, we evaluated the most promising compounds considering in vitro pharmacokinetic properties such as solubility and metabolic stability. Avoiding the phenoxy‐motif in 3‐chloro substituted compounds resulted in a four‐to‐eight‐fold increase in solubility for **18** (31.3 × 10^−6^ m) and 17 (64 × 10^−6^ m), respectively (the method is reported in Section S5.4 in the Supporting Information). The amino function in **20** only slightly increased solubility from 7.7 × 10^−6^ m of compound **4** to 13 × 10^−6^ m. In terms of metabolic stability, the trifluoromethoxy‐motif demonstrated the longest half‐life (18, *t*_1/2_ = 55 min). The stability was drastically decreased for 17 (*t*_1/2_ = 3 min), indicating the methoxy substituent to be a metabolic hotspot. Compounds **4** and **19** were moderately stable with half‐lives of 10 and 19 min, respectively. Considering pulmonary applications, moderate metabolic stability might be preferable over highly stable compounds providing a means to limit systemic exposure and risks of adverse effects.

In order to assess potential cytotoxic effects, **4** and **17** were tested against HepG2 (human liver cancer cell line) cells. QSI **4** had an LD_50_ greater than 25 × 10^−6^ m, which is acceptable considering its high activity, while compound **17** showed even less acute cellular toxicity (Table 2). Furthermore, both compounds showed no activity on hERG (human Ether‐à‐go‐go‐Related Gene) channels in a functional patch‐clamp inhibition assay (>25 × 10^−6^ m), which is an indicator for potential cardiotoxicity. Hence, avoiding this off‐target is an important prerequisite for advancing compounds toward their application in humans.^[^ ^25^ ^]^ Additionally, we performed several CYP (Cytochrome P450) inhibition assays to shed light on potential drug‐drug interactions. Compound **17** turned out to be an inhibitor of CYP2C19 (IC_50_ = 3.6 × 10^−6^ m), which could be problematic for combination with CF‐related medication.^[^ ^26^ ^]^ QSI **4** showed only slight inhibition of CYP3A4 and CYP2C9 (IC_50 _> 15 × 10^−6^ m) and no activity on the other enzymes tested (IC_50 _> 25 × 10^−6^ m). This inconspicuous profile of QSI **4** should provide sufficient latitude for combination therapy. Further analyses in the frame of a CEREP off‐target panel revealed **17** to be a binder of the human *β*2 receptor (85% binding at 10 × 10^−6^ m). This is of particular importance since this could lead to bronchospasms in our primary target organ, the lung.^[^ ^27^ ^]^ QSI **4**, on the other hand, again showed a more preferable profile (only 18% binding to the human *β*2 receptor at 10 × 10^−6^ m). The main off‐target hits of QSI **4** were dopamine transporter and norepinephrine transporter, which are located predominantly beyond the blood‐brain barrier where exposure is expected to be low. Overall, QSI **4** can be categorized as a compound with no overt findings (“red flags”) regarding in vitro safety pharmacology.

Additionally, permeation assays across a Calu‐3 monolayer were conducted in vitro. This cell line provides a representative approximation of the epithelial barrier in the bronchial pulmonary regions displaying tight‐junctions in vitro.^[^ ^28^ ^]^ Compound **17** displayed a higher epithelial permeability than QSI **4** as expressed by the higher apparent permeability parameter P_app_. However, in contrast to oral bioavailability, a lower permeability would be desired for local pulmonary applications since this might lead to an increased residence time of the active drug in the lung. For these reasons, we decided to continue our studies with QSI **4**.

### 2.4 Tolerability, Pharmacokinetics, and Target Engagement of QSI in the Murine Lung

Before starting the in vivo target‐engagement experiment, the maximum tolerated dose in mice was determined (Section S6.1, Supporting Information), which revealed that QSI **4** was well tolerated after intravenous (i.v.) administration up to a dose of 60 mg kg^−1^.

**Figure 3:**
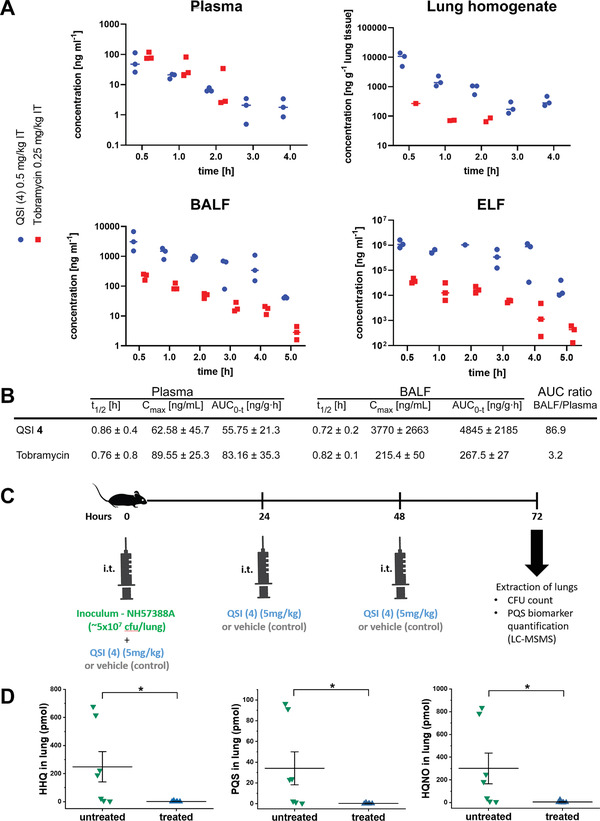
In vivo evaluation of QSI **4**. A) In vivo pharmacokinetics assessment after intratracheal instillation (*n* = 3 mice per time point per compound). B) PK parameters QSI **4** and Tob including elimination half‐life (*t*_1/2_), peak plasma concentration (*C*_max_) and area under the curve (AUC) for Plasma and ELF compartments as well as a ratio of the provided AUCs. C) Layout of the lung‐infection model. D) Absolute levels of PQS‐related biomarkers HHQ (left), PQS (middle), and HQNO (right) in infected lungs 72 h after infection in the treated and untreated groups. The compound was administered as described out in scheme (C). Unpaired *t*‐test, one‐sided *p*‐value, *n* = 7, * *p* < 0.05.

**Figure 4:**
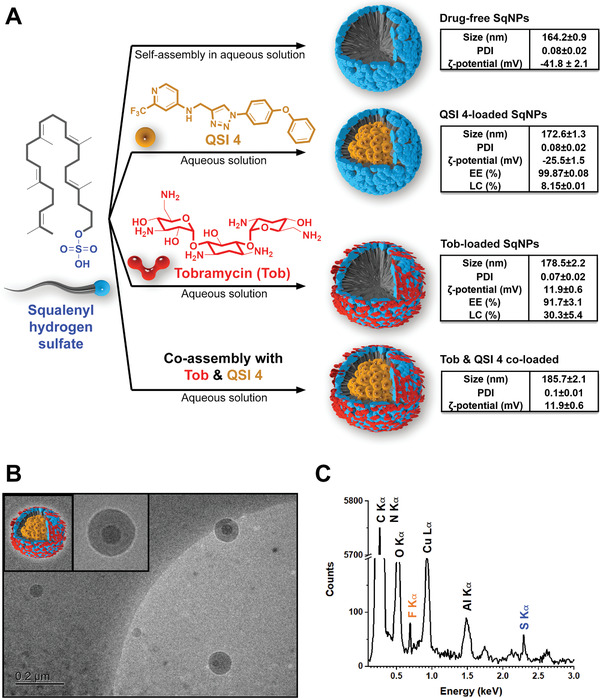
Tobramycin (Tob) and QSI **4** co‐loaded SqNPs. A) Schematic illustration of drug‐free, QSI **4**‐loaded, Tob‐loaded, and Tob and QSI **4** co‐loaded SqNPs preparation; inserted tables summarize the characteristics of the optimal nanoparticles including size, polydispersity index (PDI), *ζ*‐potential, encapsulation efficacy (EE%) and loading capacity (LC%). Three independent experiments were conducted in triplicate. B) Representative cryo‐TEM image of the Tob and QSI **4** co‐loaded SqNPs, scale bar 0.2 µm. C) Elemental composition analysis of the Tob and QSI **4** co‐loaded SqNPs using energy dispersive X‐Ray (EDX).

Moreover, the lung‐specific pharmacokinetic properties of QSI **4** were assessed. After intratracheal (IT) installation at a dose of 0.5 mg kg^−1^, QSI **4** concentrations in bronchoalveolar lavage fluid (BALF, Table S9: Supporting Information), plasma (Table S7, Supporting Information), and lung tissue (Table S8, Supporting Information) were determined at designated time points (**Figure** **3**A). Additionally, we calculated the exposure in epithelial lining fluid (ELF, Table S10, Supporting Information) from the experimental BALF parameter according to previously reported formulas (Equations S3 and S4, Supporting Information).^[^ ^29^ ^]^ Interestingly, compound **4** shows a good lung bioavailability within the time range of the PK study, especially in BALF and ELF, which can be regarded as primary target sites for treatment of PA lung infections. For instance, the half‐time (*t*_1/2_) and the maximum concentration (*C*_max_) of QSI **4** in BALF were 0.72 ± 0.5 h and 3770 ± 2663 ng mL^−1^, respectively; by contrast, plasma *C*_max_ of QSI **4** was as low as 62.62 ± 45.7 ng mL^−1^ (Figure 3B). Accordingly, the area under the curve within the experimental time‐range (AUC_0‐t_) revealed marked differences between BALF/ELF (4844 ± 2185 and 3095.83 ± 330.2 ng mL^−1^, respectively) compared to the AUC_0‐t_ of plasma (55.75 ± 21.3 ng mL^−1^). Therefore, the PK profile of QSI **4** denotes suitable lung retention in the first hours following intratracheal administration with clearly reduced systemic exposure. For comparison, we additionally investigated the PK profile of Tob using the same administration route, which showed an equivalent *t*_1/2_ in BALF (0.82 ± 0.1 h) and similarly high lung levels of Tob in the lungs compared to plasma (Section S6.2 and Tables S7 to S10 in the Supporting Information). However, exposure to BALF was lower than QSI **4**. It has been reported that aminoglycoside antibiotics are sequestered by lysosomes due to their inherent positive charges.^[^ ^30^ ^]^ Such a “lysosomal trapping” inside lung epithelial cells would explain the difference between the lung and BALF/ELF exposures, which result in the lower AUC ratio BALF/Plasma for Tob compared to QSI **4** (**Figure** **4**B). In summary, these PK assessments highlighted the prospect of administering QSI **4** directly to the pulmonary compartment and thus might provide the basis for aminoglycoside antibiotic‐QSI **4** combination therapy.

These results demonstrating high tolerability as well as suitable PK characteristics encouraged us to further investigate the pharmacology of QSI **4** in an infection mouse model (Figure 3C). Considering that QSIs do not have bactericidal activity, previously described in vivo PA lung infection models, which were primarily developed to assess the efficacy of antibiotics and almost universally use the pulmonary bacterial load (e.g., CFU) as the primary read‐out, would not be entirely suitable to test the efficacy of non‐bactericidal/non‐bacteriostatic pathoblockers like QSI **4**. We, therefore, decided to make use of a murine sub‐chronic infection model with a mucoid infection phenotype specifically enabling to assess the on‐target activity of QSIs. Mice were infected via intratracheal instillation with an inoculum of 5∙10^7^ CFU/lung of the PA isolate NH57388A possessing functional PQS QS.^[^ ^16^ ^]^ Animals were immediately treated with QSI **4** co‐administered using methyl cellulose (dose = 5 mg kg^−1^) or vehicle (control), followed by further treatments at 24 and 48 h post‐infection. The lungs were harvested at 72 h time point, homogenized and extracted using ethyl acetate. As a readout for target engagement, we quantified the signal molecule levels of HHQ, PQS, and HQNO as proximal biomarkers for the activity of the bacterial target. These signal molecules were suggested as suitable prognostic biomarkers for PA infections in CF patients.^[^ ^18^ ^]^ In addition, we measured bacterial burden in form of CFU count as a treatment endpoint (Section S6.3, Supporting Information). To our satisfaction, all auto‐inducer levels had been reduced significantly (*p* < 0.05) (Figure 3D and Table S11: Supporting Information). Moreover, CFU was reduced by four‐fold upon treatment with QSI **4** (see Table S11 in the Supporting Information). Regarding the hypothesized pharmacology of a pathoblocker target, which does not have direct bactericidal or bacteriostatic effects, this was surprising. However, considering the impact of the host immune system on bacterial clearance and the action of some PqsR‐controlled virulence factors toward immune‐system evasion (biofilm formation, pyocyanin production, elastase LasB expression), an indirect effect on the bacterial load in the lungs is plausible. However, it has to be stated that this effect is only minor compared to typical 2‐ to 4‐log unit reductions achieved by early antibiotic treatment.^[^ ^31^ ^]^

### 2.5 Tobramycin (Tob) and QSI **4** Co‐Loaded SqNP Formulation

We identified CF‐associated chronic lung infections produced by PA as a first potential indication for QSI **4**. In this regard, we considered some particular aspects of the disease as well as the treatment mode via inhalation. In the first place, even if a significant reduction of the CFU after the topical lung delivery of QSI **4** alone could be observed in the murine model, we believe that the best antibacterial effects would be achieved if the pathoblocker is delivered in combination with an approved SoC antibiotic such as Tob. Tob in solution is administered with a nebulizer twice a day (2 × 300 mg) for the management of lung infections in CF; prior to the administration of the antibiotic, the use of bronchodilators and mucolytics are often recommended to maximize the lung deposition and the mucus penetration of Tob. Altogether, the sequential inhalation of these compounds represents a heavy burden for the patient, which may ultimately reduce treatment adherence. Therefore, it would be highly desirable to simultaneously deliver QSI **4** and the antibiotic, without increasing the nebulization time of the therapy.

NP systems can be engineered to cross biological barriers, e.g., biofilms and mucus, and simultaneously deliver multiple anti‐infectives with complementary effects, thereby enhancing infection‐eradiating efficacy. We have reported the synthesis of squalenyl hydrogen sulfate, which enabled co‐assembly of both hydrophilic and hydrophobic drugs at high loading capacities – drug weight percentages – in aqueous solution.^[^ ^12^ ^]^

In this study, we applied this technology to synthesize self‐assembled Tob and QSI **4** co‐loaded squalenyl nanoparticles (SqNPs), which had a diameter of circa 200 nm and a relatively low polydispersity index (<0.2), indicative of a uniform SqNP suspension. This carrier system provided a high loading capacity for both, Tob (30.3%) and QSI **4** (8.15%). The detailed characteristics of drug‐free and drug‐loaded SqNPs are depicted in **Figure** **4**. Interestingly, a core–shell structure of the co‐loaded SqNPs could be observed by cryo‐TEM (Figure 4B), in which the localization of fluorinated QSI **4** in the core resulted in an enhanced contrast compared to that of the other organic agents. The distinctive fluorine and sulfur peaks in the elemental composition analysis (Figure 4C) further confirmed the colocalization of QSI **4** and Tob in the SqNPs nanocarrier system.

**Figure 5:**
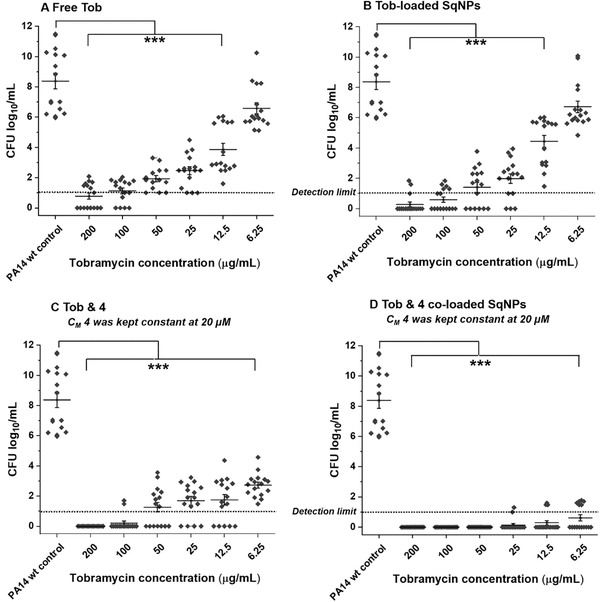
Minimum biofilm eradicating concentration (MBEC) assay on PA14 wt biofilm grown in PPGAS medium for 24 h: biofilms were treated with A) free tobramycin (Tob); B) Tob‐loaded SqNPs; C) free Tob and free **4**; D) Tob and 4 co‐loaded SqNPs. The concentration of **4** was 20 × 10^−6^ m and kept constant in all assays using **4**. After a 24 h treatment, efficacy was assessed by determination of colony‐forming units per milliliter (CFU mL^−1^). CFU mL^−1^ values are depicted logarithmically for *N* = 4, *n* = 16. Untreated PA14 wt biofilm, PA14 wt biofilms treated with either drug‐free SqNPs, free **4**, or **4**‐loaded SqNPs were served as controls (see the Supporting Information). The dotted line indicates the detection limit. Significance was calculated via One‐way ANOVA with *** indicating *p* < 0.001 versus controls.

Several preclinical and clinical studies have revealed that Tob – as the first‐line therapy – cannot fully eradicate PA‐associated infections, especially in CF patients.^[^ ^32^ ^]^ It has been reported that eDNA helps to sequester and thus inactivate Tob due to strong anionic interactions between the nucleic acid phosphate backbone and the amino functions of the aminoglycoside antibiotic.^[^ ^33^ ^]^ Since PqsR is involved in the regulation of production of PA‐borne eDNA as well as lectins, which are main biofilm matrix components, disruption of biofilm barrier integrity toward aminoglycoside treatment can be achieved by the QSI **4**. Hence, in addition to in vivo target engagement, we examined the ability of QSI **4** to enhance Tob susceptibility in PA biofilm infections. We firstly investigated the pyocyanin inhibitory efficacy of QSI **4**‐loaded SqNPs on PA strain PA14 wild type (wt). Notably, the loading of QSI **4** in SqNPs resulted in better dispersion of this hydrophobic drug in an aqueous environment and thus improved its pyocyanin inhibition by a factor of two compared to the free QSI **4** (Figure S2, Supporting Information). In the next steps, we conducted the MBEC assays on PA14 wt. As expected, Tob alone showed complete biofilm eradication at the concentrations higher than 200 µg mL^−1^ (**Figure** **5**A), which is significantly higher than that in the planktonic setting at 3.125–6.25 µg mL^−1^ (Figure S3, Supporting Information). At a Tob concentration of 6.25 µg mL^−1^, complete biofilm eradication was not at all observed in either Tob‐loaded SqNPs or free form of Tob plus QSI **4**, whereas the concentration of compound **4** was kept constant at 20 × 10^−6^ m in all treatments (Figure 5B,C). The effects of Tob were enhanced in combination with QSI **4** (20 × 10^−6^ m), resulting in lower CFUs at all treated concentrations, while the MBEC value was determined at 100 µg mL^−1^ (Figure 5C). In contrast, the Tob and QSI **4** co‐loaded SqNPs formulation was able to completely eradicate PA biofilms at a minimum Tob concentration of 6.25 µg mL^−1^ (Figure 5D). Remarkably, this concentration is in line with the upper limit of the MIC observed with the planktonic bacteria setting.

Currently, there are no clear clinical development pathways for pathoblocker approaches as well as inhaled multimodal nanomedicines. These need to be devised by actively pursued translational projects such as the presented one. In this context, one very important aspect is the widely acknowledged lack of reliable and predictive preclinical rodent models for inhaled therapies in general and chronic PA lung infections in particular.^[^ ^34^, ^35^ ^]^ This prompts researchers to make use of surrogate in vivo models or even consider moving toward solely non‐animal models. These aspects are currently being explored by us in order to propel the project further through the translational pipeline and will be reported on in future studies.

## 3 Conclusion

PA is one of the priority pathogens, for which the drug discovery and development pipeline is rather limited. Exploration of novel treatment modalities might be key to tackle the increasing threat posed by AMR. Among the various possible drug targets, PqsR is particularly attractive, as it is the essential transcriptional regulator of the PQS QS system serving major functions in PA virulence. It holds potential for avoiding the generation of bacterial resistance as well as compatibility with aminoglycoside (Tob) backbone treatment. Blocking this receptor results in affecting a number of down‐stream regulated processes such as virulence‐factor production, e.g., pyocyanin, biofilm formation or eDNA secretion. By this means, pathoblockers support the host immune system as well as potentiate co‐administered antibiotics in order to effectively eradicate the infection.

In this study, we successfully conducted a hit‐to‐lead campaign resulting in a novel PqsR QSI class. In order to enable facile compound diversification, we established a divergent synthesis of a novel PqsR QSI class. We were able to solve the crystal structure of PqsR in complex with QSI **4**. It is worth mentioning that we observed the opening of an additional small pocket upon the exploitation of a growth vector in the Eastern part of the molecule, which explains the observed non‐linear SAR.

Optimized lead QSI **4** demonstrated high potency, suitable PK for pulmonary applications, a favorable safety pharmacology profile and was well tolerated in mice. Although CEREP off‐target paneling did not raise immediate “red flags” for translation of QSI **4**, the effects on mentioned CNS‐related neurotransporters will be considered in our efforts, during in‐depth PK/PD assessments, and extended in vivo safety studies, which have to be conducted alongside preclinical development.

In a murine lung‐infection model with a mucoid PA isolate, we successfully demonstrated the target engagement of the QSI **4** by recording a significant reduction of the proximal biomarkers HHQ, PQS, and HQNO. Besides, even though this was not expected from an anti‐virulence compound, a four‐fold reduction of the bacterial load was observed.

The recent failures in the development of inhaled antibiotics for bronchiectasis highlight the challenges for the translation of novel anti‐pseudomonal drugs. The typically complex and multimodal treatment regimens applied in CF patients point toward further exploring the potential synergistic drug combinations. To this end, novel technologies for efficient co‐delivery will be needed to ensure/facilitate patient compliance.^[^ ^32^ ^]^ We generated a tailor‐made nanocarrier formulation suitable for a co‐delivery of our QSI and SoC antibiotic Tob. The nanoformulation not only improves the water solubility of QSI **4** but also showed remarkable efficacy in biofilm eradication, which may therefore enable the simultaneous delivery of the compound and Tob via inhalation. Moreover, inhalation is painless, minimally invasive,^[^ ^35^ ^]^ avoids the first‐pass metabolism and provides instant drug delivery to the organ of action, the lung. This could ultimately result in better lung disposition of the combination therapy and reduction of systemic side effects.^[^ ^35^ ^]^

In summary, this work represents an important step in a highly translational approach of PqsR‐targeting QSIs and provides the basis for the development of a novel adjunctive treatment option against PA infections.

## 4 Experimental Section

Materials and experimental details are provided in the Supporting Information.

Mouse experiments were approved by the Landesamt für Soziales, Gesundheit und Verbraucherschutz of the State of Saarland in accordance with the national guidelines for animal treatment on August, 17th, 2017 (22/2017).

## Conflict of Interest

The authors A. S. A. Ahmed, M. Empting, M. Hamed, R. W. Hartmann, J. Haupenthal, T. Hesterkamp, A. A. M. Kamal, C. K. Maurer, T. Röhrig, C. Schütz, S. Yahiaoui, M. Zender have filed patent WO2020007938 (EP18181475) “PqsR Inverse Agonists”, which claims intellectual property for the structures published herein.

## Supporting information

Supporting Information

## Data Availability

Research data are not shared.

## References

[1] S. Karakonstantis , E. I. Kritsotakis , A. Gikas , J. Antimicrob. Chemother. 2020, 75, 271. 31586417 10.1093/jac/dkz401

[2] J. S. Talwalkar , T. S. Murray , Clin. Chest Med. 2016, 37, 69. 26857769 10.1016/j.ccm.2015.10.004

[3] a) S. Palser , S. Smith , E. F. Nash , A. Agarwal , A. R. Smyth , Cochrane Database Syst. Rev. 2019, 12, CD012300; 31845758 10.1002/14651858.CD012300.pub2 PMC6916140

[4] a) WHO , “https://www.who.int/medicines/publications/global‐priority‐list‐antibiotic‐resistant‐bacteria/en/” (accessed: November 2020);

[5] S. Wagner , R. Sommer , S. Hinsberger , C. Lu , R. W. Hartmann , M. Empting , A. Titz , J. Med. Chem. 2016, 59, 5929. 26804741 10.1021/acs.jmedchem.5b01698

[6] a) S. Mühlen , P. Dersch , Curr. Top. Microbiol. Immunol. 2016, 398, 147; 26942418 10.1007/82_2015_490

[7] H. Cao , G. Krishnan , B. Goumnerov , J. Tsongalis , R. Tompkins , L. G. Rahme , Proc. Natl. Acad. Sci. USA 2001, 98, 14613. 11724939 10.1073/pnas.251465298 PMC64730

[8] a) E. Déziel , S. Gopalan , A. P. Tampakaki , F. Lépine , K. E. Padfield , M. Saucier , G. Xiao , L. G. Rahme , Mol. Microbiol. 2005, 55, 998; 15686549 10.1111/j.1365-2958.2004.04448.x

[9] A. A. M. Kamal , L. Petrera , J. Eberhard , R. W. Hartmann , Org. Biomol. Chem. 2017, 15, 4620. 28513746 10.1039/c7ob00263g

[10] C. Schütz , M. Empting , Beilstein J. Org. Chem. 2018, 14, 2627. 30410625 10.3762/bjoc.14.241 PMC6204780

[11] a) A. Thomann , A. G. G. de Mello Martins , C. Brengel , M. Empting , R. W. Hartmann , ACS Chem. Biol. 2016, 11, 1279; 26882081 10.1021/acschembio.6b00117

[12] D.‐K. Ho , X. Murgia , C. de Rossi , R. Christmann , A. G. Hüfner de Mello Martins , M. Koch , A. Andreas , J. Herrmann , R. Müller , M. Empting , R. W. Hartmann , D. Desmaele , B. Loretz , P. Couvreur , C.‐M. Lehr , Angew. Chem., Int. Ed. 2020, 59, 10292. 10.1002/anie.202001407 PMC7317969 32243047

[13] R. D. Moore , C. R. Smith , P. S. Lietman , J. Infect. Dis. 1984, 149, 443. 6715900 10.1093/infdis/149.3.443

[14] a) V. Badha , R. Moore , J. Heffernan , P. Castaneda , A. McLaren , D. Overstreet , J. Bone Joint Infect. 2019, 4, 1; 10.7150/jbji.29711 PMC6367195 30755841

[15] M. Zender , F. Witzgall , A. Kiefer , B. Kirsch , C. K. Maurer , A. M. Kany , N. Xu , S. Schmelz , C. Börger , W. Blankenfeldt , M. Empting , ChemMedChem 2019, 15, 188. 31709767 10.1002/cmdc.201900621 PMC7004148

[16] X. Murgia , A. M. Kany , C. Herr , D.‐K. Ho , C. de Rossi , R. Bals , C.‐M. Lehr , A. K. H. Hirsch , R. W. Hartmann , M. Empting , T. Rohring , Sci. Rep. 2020, 10, 16502. 33020513 10.1038/s41598-020-73459-5 PMC7536435

[17] a) A. Lorenz , V. Pawar , S. Häussler , S. Weiss , FEBS Lett. 2016, 590, 3941; 27730639 10.1002/1873-3468.12454

[18] H. L. Barr , N. Halliday , D. A. Barrett , P. Williams , D. L. Forrester , D. Peckham , K. Williams , A. R. Smyth , D. Honeybourne , J. L. Whitehouse , E. F. Nash , J. Dewar , A. Clayton , A. J. Knox , M. Cámara , A. W. Fogarty , J. Cystic Fibrosis 2017, 16, 230. 10.1016/j.jcf.2016.10.005 PMC5345566 27773591

[19] D. J. Abraham , A. Burger , Gale Virtual Reference Library, John Wiley and Sons, Hoboken, NJ 2003.

[20] a) T. E. Ballard , J. J. Richards , A. L. Wolfe , C. Melander , Chemistry 2008, 14, 10745; 18942682 10.1002/chem.200801419

[21] M. Starkey , F. Lepine , D. Maura , A. Bandyopadhaya , B. Lesic , J. He , T. Kitao , V. Righi , S. Milot , A. Tzika , L. Rahme , PLoS Pathog. 2014, 10, 1004321. 10.1371/journal.ppat.1004321 PMC4140854 25144274

[22] F. Soukarieh , R. Liu , M. Romero , S. N. Roberston , W. Richardson , S. Lucanto , E. V. Oton , N. R. Qudus , A. Mashabi , S. Grossman , S. Ali , T. Sou , I. Kukavica‐Ibrulj , R. C. Levesque , C. A. S. Bergström , N. Halliday , S. N. Mistry , J. Emsley , S. Heeb , P. Williams , M. Cámara , M. J. Stocks , Front. Chem. 2020, 8, 204. 32432073 10.3389/fchem.2020.00204 PMC7213079

[23] a) M. F. Richter , P. J. Hergenrother , Ann. N. Y. Acad. Sci. 2019, 1435, 18; 29446459 10.1111/nyas.13598 PMC6093809

[24] M. J. Waring , J. Arrowsmith , A. R. Leach , P. D. Leeson , S. Mandrell , R. M. Owen , G. Pairaudeau , W. D. Pennie , S. D. Pickett , J. Wang , O. Wallace , A. Weir , Nat. Rev. Drug Discovery 2015, 14, 475. 26091267 10.1038/nrd4609

[25] M. C. Sanguinetti , M. Tristani‐Firouzi , Nature 2006, 440, 463. 16554806 10.1038/nature04710

[26] C. L. Jordan , T. L. Noah , M. M. Henry , Pediatr. Pulmonol. 2016, 51, S61. 27662106 10.1002/ppul.23505

[27] R. V. Lewis , C. Lofthouse , Drug Saf. 1993, 9, 272. 7903148 10.2165/00002018-199309040-00005

[28] a) C. I. Grainger , L. L. Greenwell , D. J. Lockley , G. P. Martin , B. Forbes , Pharm. Res. 2006, 23, 1482; 16779708 10.1007/s11095-006-0255-0

[29] S. Kiem , J. J. Schentag , Antimicrob. Agents Chemother. 2008, 52, 24. 17846133 10.1128/AAC.00133-06 PMC2223903

[30] D. Beauchamp , P. Gourde , M. Simard , M. G. Bergeron , Antimicrob. Agents Chemother. 1992, 36, 2204. 1444301 10.1128/aac.36.10.2204 PMC245477

[31] C. Cigana , S. Ranucci , A. Rossi , I. de Fino , M. Melessike , A. Bragonzi , Eur. Respir. J. 2020, 55, 1802456. 31624114 10.1183/13993003.02456-2018 PMC7057181

[32] D.‐K. Ho , B. L. B. Nichols , K. J. Edgar , X. Murgia , B. Loretz , C.‐M. Lehr , Eur. J. Pharm. Biopharm. 2019, 144, 110. 31493510 10.1016/j.ejpb.2019.09.002

[33] W.‐C. Chiang , M. Nilsson , P. Ø. Jensen , N. Høiby , T. E. Nielsen , M. Givskov , T. Tolker‐Nielsen , Antimicrob. Agents Chemother. 2013, 57, 2352. 23478967 10.1128/AAC.00001-13 PMC3632962

[34] a) K. Lewis , Nat. Rev. Drug Discovery 2013, 12, 371; 23629505 10.1038/nrd3975

[35] D. Movia , A. Prina‐Mello , Animals 2020, 10, 1259. 10.3390/ani10081259 PMC7460012 32722259

