# Supplementary material for: A New PqsR Inverse Agonist Potentiates Tobramycin Efficacy to Eradicate Pseudomonas aeruginosa Biofilms
Source: Adv Sci (Weinh). 2021 Mar 18;8(12):2004369. doi: 10.1002/advs.202004369 (PMC8224453; doi:10.1002/advs.202004369)
Supplement: Supplementary file 1 — Supporting Information [file ADVS-8-2004369-s001.pdf]

© 2021 Wiley-VCH GmbH

ADVANCED SCIENCE
Open Access

Supporting Information

for *Adv. Sci.,* DOI: 10.1002/advs.202004369

A New PqsR Inverse Agonist Potentiates Tobramycin Efficacy to Eradicate *Pseudomonas aeruginosa* Biofilms

*Christian Schütz, Duy-Khiet Ho, Mostafa Mohamed Hamed, Ahmed Saad Abdelsamie, Teresa Röhrig, Christian Herr, Andreas Martin Kany, Katharina Rox, Stefan Schmelz, Lorenz Siebenbürger, Marius Wirth, Carsten Börger, Samir Yahiaoui, Robert Bals, Andrea Scrima, Wulf Blankenfeldt, Justus Constantin Horstmann, Rebekka Christmann, Xabier Murgia, Marcus Koch, Aylin Berwanger, Brigitta Loretz, Anna Katharina Herta Hirsch, Rolf Wolfgang Hartmann, Claus-Michael Lehr, and Martin Empting**

© Copyright 2021. WILEY-VCH GmbH.

Supporting Information

A new PqsR inverse agonist potentiates tobramycin efficacy to eradicate Pseudomonas aeruginosa biofilms

*Christian Schütz, Duy-Khiet Ho, Mostafa M. Hamed, Ahmed S. Abdelsamie, Teresa Röhrig, Christian Herr, Andreas M. Kany, Katharina Rox, Stefan Schmelz, Lorenz Siebenbürger, Marius Wirth, Carsten Börger, Samir Yahiaoui, Robert Bals, Andrea Scrima, Wulf Blankenfeldt, Justus C. Horstmann, Rebekka Christmann, Xabier Murgia, Marcus Koch, Aylin Berwanger, Brigitta Loretz, Anna K. H. Hirsch, Rolf W. Hartmann, Claus Michael Lehr and Martin Empting**

Contents

1. Chemistry 1
 1.1. General procedure 1: Di-Boc-protection 1
 1.2. General procedure 2: Mono-Boc-deprotection 1
 1.3. General procedure 3: Propargylation 2
 1.4. General procedure 4: Boc-Deprotection 2
 1.5. General procedure 5: Synthesis of 1,2,3-triazoles, modified from Barral *et al.*[1] 2
 1.6. Synthesis of compounds **1**-**20** 3
2. Nanoparticle Preparation and Characterization 12
3. Primary Activity Assays 15
 3.1. Reporter Gene Assay 15
 3.2. Pyocyanin assay 15
4. Secondary Activity Assays 17
 4.1. Alkylquinolone Inhibition Assay 17
 4.2. MIC Assay 18
 4.3. Minimum Biofilm Eradicating Concentration (MBEC) assay 19
5. *In vitro* ADME/T and Safety Pharmacology 22
 5.1. CEREP off-targets assay 22
 5.2. CYP and hERG inhibition 23
 5.3. Cytotoxicity Assays 23
 5.4. Kinetic Turbidimetric Solubility Assay 23

5.5. Metabolic stability 23
5.6. Permeability studies 24
6. *In vivo* Experiments 25
6.1. Tolerability of QSI (**4**) and SqNP-assembly in Mice 25
A) Mouse MTD of QSI **4**. 25
B) Tolerability QSI-loaded SqNP after intracheal administration. 25
6.2. PK Sample Preparation and Analysis 27
6.3. Design of *in vivo* Target Engagement Study 31
7. Xray Crystallography 34
7.1. Expression and Purification of $PqsR^{91-319}$ 34
7.2. Protein Crystallization, Data Collection and Structure Solution 34
8. Analysis and Visualization of complex structures of compounds **2** and **4** bound to $PqsR^{91-319}$ 37
9. Statistics 38
10. References 40

1. Chemistry

Solvents and chemicals were used from Zentrales Chemikalienlager der Universität des Saarlandes or specified vendors (Carbolution, Acros Organics, Sigma Aldrich, Fluorochem, TCI, abcr, Alfa Aesar, VWR). For automated flash chromatography, either a Teledyne ISCO CombiFlash Rf+ 150 or a Teledyne ISCO CombiFlash NEXTGEN 300+ equipped with RediSepRf silica columns were used. NMR spectra were recorded on either a Bruker 300 MHz or a Bruker UltraShield Plus 500 MHz device. Chemical shifts (δ) were given in parts per million (ppm) and referenced against the residual solvent peak. Coupling constants (J) were given in Hertz (Hz). Multiplicities were described using the abbreviations s (singlet), bs (broad singlet), d (doublet), dd (doublet of a doublet), ddd (doublet of a doublet of a doublet), t (triplet), dt (doublet of a triplet), q (quartet), hept (heptet) and m (multiplet). Liquid chromatography-mass spectrometry (LC-MS) spectra were measured on a Thermo Scientific DIONEX UltiMate3000 consisting of a diode array detector, column compartment, autosampler and pump. High resolution mass (HRMS) was determined by LC-MS/MS using Thermo Scientific Q Exactive Focus Orbitrap LC-MS/MS system. General procedures of chemical synthesis are described as follows:

1.1. General procedure 1: Di-Boc-protection

Synthesis of **IIa**, **IIb** described in Scheme S1 (see below).

To a solution of the corresponding 2-trifluoromethyl-4-aminopyridine (20.0 mmol) in DCM (60 mL) were added Boc_2O (9.82 g, 45.0 mmol), DIPEA (7.66 mL, 44.0 mmol) and DMAP (806 mg, 6.60 mmol). The reaction mixture was stirred for 18 h at r.t., and saturated NH_4Cl solution was added followed by extraction with EtOAc. The combined organic layers were washed with Brine, dried over Na_2SO_4, filtrated and evaporated under reduced pressure. The crude product was used without any further purification.

1.2. General procedure 2: Mono-Boc-deprotection

Synthesis of **IIIa**, **IIIb** described in Scheme S1 (see below).

The corresponding di-*tert*-butyl(2-(trifluoromethyl)pyridin-4-yl)dicarbamate (6.00 mmol) was dissolved in DCM/TFA 9:1 (12 mL) and stirred for 16 h at r.t. until the starting material was fully consumed (TLC control). Saturated Na_2CO_3 solution was added followed by extraction with DCM. The combined organic layers were dried over Na_2SO_4 filtrated and concentrated *in vacuo*. The desired compound was used without further purification.

1.3. General procedure 3: Propargylation

Synthesis of **IVa**, **IVb** described in Scheme S1 (see below).

A solution of the corresponding *tert*-butyl(2-(trifluoromethyl)pyridin-4-yl)carbamate (5.70 mmol) was cooled to 0 °C and NaH (60 wt%) (273 mg, 6.83 mmol) was added portionwise under vigorous stirring. After 30 min the reaction mixture was warmed to r.t., and propargylbromide (0.56 mL, 7.40 mmol) was added dropwise and stirred for 3 h at r.t. Addition of saturated NH_4Cl solution and extraction with Et_2O followed by washing the combined organic layers with saturated LiCl solution yielded the crude product after drying over Na_2SO_4, filtration and concentration *in vacuo*, which was purified by automated column chromatography using a gradient of PE/EtOAc.

1.4. General procedure 4: Boc-Deprotection

Synthesis of **Va**, **Vb** described in Scheme S1 (see below).

The corresponding *tert*-butyl prop-2-yn-1-yl(2-(trifluoromethyl)pyridin-4-yl)carbamate (4.03 mmol) was dissolved in DCM (1 mL) and TFA (1 mL) was added. After stirring at r.t. for 18 h, the mixture was cooled to 0 °C, and saturated Na_2CO_3 solution was added. The reaction mixture was extracted with DCM, and the combined organic layers were washed with Brine, dried over Na_2SO_4, filtered and concentrated under reduced pressure. Automated flash chromatography using a gradient of PE/EtOAc afforded the title compound.

1.5. General procedure 5: Synthesis of 1,2,3-triazoles, modified from Barral *et al.*[1]

Synthesis of **compounds 1-19, VI** is described in Scheme S1 (see below).

To a solution of the corresponding aniline (0.100 mmol) in MeCN (0.1 mL/mmol), *tert*-butyl nitrite (0.110 mmol, 12.6 mg, 14.5 µL) was added at 0 °C. After stirring for 5 min, trimethylsilylazide (0.110 mmol, 13.3 mg, 15.2 µL) was slowly added. The mixture was stirred for 2 h, and *N*-(prop-2-yn-1-yl)-2-(trifluoromethyl)pyridine-4-amine (**E**) (0.120 mmol, 24.0 mg) was added in one portion. After addition of DIPEA (0.120 mmol, 15.5 mg, 20.9 µL), the reaction mixture was purged with argon, and $CuSO_4 \cdot 5\,H_2O$ (10 mol%) and Na-ascorbate (20 mol%) were added. The mixture was again purged with argon and stirred at r.t. LCMS analysis indicated full conversion after 1 h, and saturated NH_4Cl solution was added to the reaction mixture and stirred for 5 min. Then EtOAc and saturated EDTA solution were added, and the mixture was stirred for 10 min until the aqueous phase showed a clear blue color. The phases were separated, and the aqueous phase was extracted with EtOAc three times. The combined organic layers were dried over $MgSO_4$, filtered and concentrated *in vacuo*. The crude product was purified via automated flash chromatography using a gradient of PE/EtOAc.

1.6. Synthesis of compounds 1-20

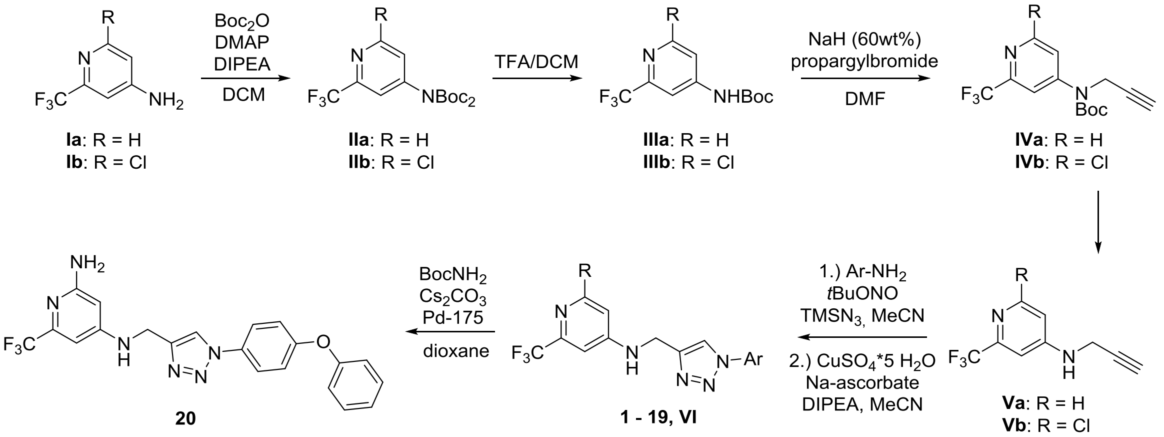

*Scheme S1. General schematic procedures and detailed reaction conditions for the synthesis of compound **1-20**.*

Characterization of *tert*-butyl (*tert*-butoxycarbonyl)(2-(trifluoromethyl)pyridin-4-yl)dicarbamate (**IIa**): 2-trifluoromethyl-4-aminopyridine (**Ia**) was used in the reaction and yielded 7.21 g (19.9 mmol, >98%) of the desired product as colorless crystals.

LC-MS: m/z: 363 $(M+H)^+$

^{1}H-NMR (300 MHz, $CDCl_3$): δ [ppm] = 8.72 (d, *J* = 5.4 Hz, 1 H), 7.52 (d, *J* = 1.7 Hz, 1 H), 7.31 (dd, *J* = 5.3 Hz, *J* = 1.8 Hz, 1 H), 1.47 (s, 18 H).
^{13}C-NMR (76 MHz, $CDCl_3$): δ [ppm] = 150.8, 150.3, 148.1, 124.2, 122.5, 119.0, 118.1, 84.53, 27.78.

Characterization of *tert*-butyl (2-(trifluoromethyl)pyridin-4-yl)carbamate (**IIIa**): di-*tert*-butyl(2-(trifluoromethyl)pyridin-4-yl)dicarbamate (**IIa**) was used in the reaction and afforded the desired product as colorless crystals (1.57 g, 5.98 mmol, >98%).
LC-MS: m/z: 207 (M-*t*Bu+H)$^+$
^{1}H-NMR (300 MHz, $CDCl_3$): δ [ppm] = 8.53 (d, *J* = 5.6 Hz, 1 H), 7.78 (d, *J* = 2.0 Hz, 1 H), 7.44 (dd, *J* = 5.5 Hz, *J* = 2.1 Hz, 1 H), 6.92 (bs, 1 H), 1.53 (s, 9 H).
^{13}C-NMR (76 MHz, $CDCl_3$): δ [ppm] = 151.4, 150.8, 146.9, 133.2), 114.2, 109.3, 109.2, 82.48, 28.16.

Characterization of *tert*-butyl prop-2-yn-1-yl(2-(trifluoromethyl)pyridin-4-yl)carbamate (**IVa**) According to general procedure 3, *tert*-butyl (2-(trifluoromethyl)pyridin-4-yl)carbamate (**IIIa**) was used in the reaction and yielded 1.56 g (5.18 mmol, 91%) of a slight yellow solid.
LC-MS: m/z: 245 (M-*t*Bu +H)$^+$
^{1}H-NMR (300 MHz, $CDCl_3$): δ [ppm] = 8.63 (d, *J* = 5.5, 1 H), 7.85 (d, *J* = 2.0 Hz, 1 H), 7.55 (dd, *J* = 5.5 Hz, *J* = 2.1 Hz, 1 H), 4.48 (d, *J* = 2.3 Hz, 2 H), 2.25 (t, *J* = 2.4 Hz, 1 H).
^{13}C-NMR (75 MHz, $CDCl_3$): δ [ppm] = 151.8, 150.2, 150.1, 118.8, 114.5, 114.4, 114.3, 83.32, 78.04, 72.81, 38.01, 27.77.

Characterization of *N*-(prop-2-yn-1-yl)-2-(trifluoromethyl)pyridine-4-amine (**Va**): *tert*-butyl prop-2-yn-1-yl(2-(trifluoromethyl)pyridin-4-yl)carbamate (**IVa**) was used in the reaction and afforded the title compound as a white solid 802 mg (4.01 mmol, >98%).
LC-MS: m/z: 201 (M+H)$^+$
^{1}H-NMR (300 MHz, $CDCl_3$): δ [ppm] = 8.31 (d, *J* = 6.5 Hz, 1 H), 7.72 (bs, 1 H), 7.16 (d, *J* = 2.1 Hz, 1 H), 6.89 (dd, *J* = 6.5 Hz, *J* = 2.3 Hz, 1 H), 4.10 (d, *J* = 2.4 Hz, 2 H), 2.33 (t, *J* = 2.6 Hz, 1 H).
^{13}C-NMR (75 MHz, $CDCl_3$): δ [ppm] = 156.7, 145.2, 143.7, 119.33, 109.40, 106.50, 73.80, 73.63, 32.75.

Characterization of *tert*-butyl (*tert*-butoxycarbonyl)(2-chloro-6-(trifluoromethyl)pyridin-4-yl)carbamate (**IIb**): 2-chloro-6-(trifluoromethyl)pyridin-4-amine (**Ib**) was used in the reaction and yielded 7.32 g (19.8 mmol, >98%) of the desired product as colorless crystals.

LC-MS: m/z: 397 $(M+H)^+$

Characterization of *tert*-butyl (2-chloro-6-(trifluoromethyl)pyridin-4-yl)carbamate (**IIIb**): *tert*-butyl (*tert*-butoxycarbonyl)(2-chloro-6-(trifluoromethyl)pyridin-4-yl)carbamate (**IIb)** was used in the reaction and afforded the desired product as colorless crystals (1.72 g, 5.82 mmol, 97%).

LC-MS: m/z: 240 $(M+H\text{-}t\text{Bu})^+$

^{1}H-NMR (300 MHz, $CDCl_3$): δ [ppm] = 1.54 (s, 9 H), 6.99 (bs, 1 H), 7.61 – 7.62 (m, 1 H), 7.63 – 7.64 (m, 1 H).

^{13}C-NMR (76 MHz, $CDCl_3$): δ [ppm] = 28.08, 83.01, 108.4, 114.2, 118.8, 122.5, 148.7, 151.2, 171.3.

Characterization of *tert*-butyl (2-chloro-6-(trifluoromethyl)pyridin-4-yl)(prop-2-yn-1-yl)carbamate (**IVb**): *tert*-butyl (2-chloro-6-(trifluoromethyl)pyridin-4-yl)carbamate (**IIIb**) was used in the reaction and yielded 1.83 g (5.47 mmol, 96%) of a slight yellow solid.

LC-MS: m/z: 279 $(M+H\text{-}t\text{Bu})^+$

^{1}H-NMR (300 MHz, $CDCl_3$): δ [ppm] = 1.47 (s, 9 H), 2.17 – 2.18 (m, 1 H), 4.60 (bs, 2 H), 8.01 – 8.03 (m, 1 H), 8.66 – 8.68 (m, 1 H).

Characterization of 2-Chloro-*N*-(prop-2-yn-1-yl)-6-(trifluoromethyl)pyridin-4-amine (**Vb**): *tert*-butyl (2-chloro-6-(trifluoromethyl)pyridin-4-yl)(prop-2-yn-1-yl)carbamate (**IVb**) was used in the reaction and afforded the title compound as a white solid 938 mg (4.00 mmol, >98%).

LC-MS: m/z: 235 $(M+H)^+$

^{1}H-NMR (300 MHz, acetone-d_6): δ [ppm] = 7.09 (d, J = 2.0 Hz, 1 H), 7.03 (bs, 1 H), 6.87 (d, J = 2.0 Hz, 1 H), 4.19 (dd, J = 6.0 Hz, J = 2.6 Hz, 2 H), 2.88 (bs, 2 H), 2.82. (t, J = 2.5 Hz, 1 H).

^{13}C-NMR (76 MHz, acetone-d_6): δ [ppm] = 159.3, 156.7, 152.3, 122.8, 109.7, 105.8, 79.27, 73.12, 32.05

Characterization of *N*-((1-(2-fluorophenyl)-1*H*-1,2,3-triazol-4-yl)methyl)-2-(trifluoromethyl)pyridin-4-amine (compound **6**): according to the general procedure 5, 2-fluoroaniline was used in the reaction and yielded 30.2 mg (89.5 µmol, 86%) of a slightly yellow solid.

^{1}H-NMR (500 MHz, $CDCl_3$): δ [ppm] = 8.35 (bs, 1 H), 8.19 (bs, 1 H), 7.9 – 7.94 (m, 1 H), 7.48 – 7.44 (m, 1 H), 7.36 – 7.31 (m, 2 H), 6.97 (bs, 1 H), 6.72 (bs, 1 H), 5.43 (bs, 1 H), 4.64 (bs, 2 H).

^{13}C-NMR (126 MHz, $CDCl_3$): δ [ppm] = 154.1, 152.1, 153.3, 116.7, 116.9, 124.5, 125.1, 130.3, 38.14.

HRMS (ESI): $C_{15}H_{12}F_4N_5^+$ $(M+H)^+$ calculated: 338.10234 found: 338.10150

Characterization of *N*-((1-(3-fluorophenyl)-1*H*-1,2,3-triazol-4-yl)methyl)-2-(trifluoromethyl)pyridin-4-amine (compound **5**): according to the general procedure 5, 3-fluoroaniline was used in the reaction and yielded 31.8 mg (94.2 µmol, 94%) of a yellow solid.

^{1}H-NMR (500 MHz, $CDCl_3$): δ [ppm] = 8.34 (bs, 1 H), 8.02 (bs, 1 H), 7.52 – 7.49 (m, 3 H), 7.17 (bs, 1 H), 6.95 (bs, 1 H), 6.70 (bs, 1 H), 5.38 (bs, 1 H), 4.63 (bs, 2 H).

^{13}C-NMR (126 MHz, $CDCl_3$): δ [ppm] = 164.4, 162.4, 153.8, 138.4, 131.7, 116.4, 116.2, 116.1, 108.8, 108.5, 38.70.

HRMS (ESI): $C_{15}H_{12}F_4N_5^+$ $(M+H)^+$ calculated: 338.10234 found: 338.10144

Characterization of *N*-((1-(4-fluorophenyl)-1*H*-1,2,3-triazol-4-yl)methyl)-2-(trifluoromethyl)pyridin-4-amine (compound **3**): according to the general procedure 5, 4-fluoroaniline (11.1 mg, 0.100 mmol) was used in the reaction and yielded 31.8 mg (94.3 µmol, 27%) of a yellow solid.

^{1}H-NMR (500 MHz, acetone-d_6): δ [ppm] = 8.66 (bs, 1 H), 8.31 (bs, 1 H), 7.94 – 7.91 (m, 2 H), 7.39 – 7.35 (m, 2 H), 7.20 (bs, 1H), 6.92 (bs, 1 H), 6.83 (bs, 1 H), 4.66 (bs, 2 H).

^{13}C-NMR (126 MHz, acetone-d_6): δ [ppm] = 163.5, 161.6, 154.8, 122.9, 122.8, 117.1, 116.8, 38.20.

HRMS (ESI): $C_{15}H_{12}F_4N_5^+$ $(M+H)^+$ calculated: 338.10234 found: 338.10151

Characterization of *N*-((1-(2-chlorophenyl)-1*H*-1,2,3-triazol-4-yl)methyl)-2-(trifluoromethyl)pyridin-4-amine (compound **7**): according to the general procedure 5, 2-

chloroaniline (12.8 mg, 0.100 mmol) was used in the reaction and yielded 9.36 mg (26.5 µmol, 94%) of a colorless solid.

^{1}H-NMR (500 MHz, acetone-d_6): δ [ppm] = 8.36 (bs, 1 H), 8.23 (bs, 1 H), 7.71 (dd, *J* = 7.8 Hz, *J* = 1.7 Hz, 1 H), 7.66 – 7.64 (m, 1 H), 7.61 – 7. 63 (m, 1 H), 7.60– 7.57 (m, 1 H), 7.14 (bs, 1 H), 6.92 – 6.90 (m, 1 H), 6.86 (bs, 1 H), 4.70 (d, *J* = 5.7 Hz, 2 H).

^{13}C-NMR (126 MHz, acetone-d_6): δ [ppm] = 154.9, 135.6, 131.7, 131.0, 129.2, 128.7, 128.5, 38.15.

HRMS (ESI): $C_{15}H_{12}ClF_3N_5^+$ (M+H)$^+$ calculated: 354.07278 found: 354.07205

Characterization of *N*-((1-(3-chlorophenyl)-1*H*-1,2,3-triazol-4-yl)methyl)-2-(trifluoromethyl)pyridin-4-amine (compound **8**): according to the general procedure 5, 3-chloroaniline was used in the reaction and yielded 30.1 mg (85.1 µmol, 85%) of a colorless solid.

^{1}H-NMR (500 MHz, acetone-d_6): δ [ppm] = 8.64 (s, 1 H), 8.22 (d, *J* = 5.7 Hz, 1 H), 7.95 (dd, *J* = 2.0 Hz, 1 H), 7.88 – 7.886(m, 1 H), 7.61 (dd, *J* = 8.1 Hz, 1 H), 7.52 – 7.50 (m, 1 H), 7.13 (d, *J* = 2.1 Hz, 1 H), 6.88 (dd, *J* = 5.7 Hz, *J* = 2.3 Hz, 1 H), 6.83 (bs, 1 H), 4.68 (d, *J* = 5.8 Hz, 2 H).

^{13}C-NMR (126 MHz, acetone-d_6): δ [ppm] = 154.9, 150.5, 138.6, 153.2, 131.8, 128.8, 121.1, 120.4, 118.9, 109.4, 104.9, 38.23.

HRMS (ESI): $C_{15}H_{12}ClF_3N_5^+$ (M+H)$^+$ calculated: 354.07278 found: 354.07213

Characterization of *N*-((1-(4-chlorophenyl)-1*H*-1,2,3-triazol-4-yl)methyl)-2-(trifluoromethyl)pyridin-4-amine (compound **9**): according to the general procedure 5, 4-chloroaniline was used in the reaction and yielded 24.4 mg (68.9 µmol, 69%) of an off-white solid.

^{1}H-NMR (500 MHz, acetone-d_6): δ [ppm] = 8.58 (s, 1 H), 8.22 (d, *J* = 5.7 Hz, 1 H), 7.93 – 7.90 (m, 2 H), 7.63 – 7.60 (m, 2 H), 7.12 (d, *J* = 2.4 Hz, 1 H), 6.88 (dd, *J* = 7.9 Hz, *J* = 2.3 Hz, 1 H), 6.82 (bs, 1 H), 4.67 (d, *J* = 5.8 Hz, 2 H).

^{13}C-NMR (126 MHz, acetone-d_6): δ [ppm] = 154.9, 150.5, 146.1, 136.3, 133.9, 130.2, 122.1, 121.1, 109.5, 104.9, 38.23.

HRMS (ESI): $C_{15}H_{12}ClF_3N_5^+$ (M+H)$^+$ calculated: 354.07278 found: 354.07220

Characterization of 4-(4-(((2-(trifluoromethyl)pyridin-4-yl)amino)methyl)-1*H*-1,2,3-triazol-1-yl)phenol (compound **10**): the compound was prepared according to the general procedure 5. 4-Aminophenol (10.9 g, 0.100 mmol) was used in the reaction and automated flash chromatography (PE/EtOAc) afforded the title compound as a yellow solid (11.5 mg, 34.3 µmol, 34%).

HRMS (ESI): $C_{15}H_{13}F_3N_5O^+$ $(M+H)^+$ calculated: 336.10667 found: 336.10596

Characterization of *N*-((1-(4-methoxyphenyl)-1*H*-1,2,3-triazol-4-yl)methyl)-2-(trifluoromethyl)pyridin-4-amine (compound **11**): according to the general procedure 5, 4-methoxyaniline was used in the reaction and yielded 11.2 mg (32.1 µmol, 32%) of a colorless solid.

^{1}H-NMR (500 MHz, acetone-d_6): δ [ppm] = 8.44 (s, 1 H), 8.22 (d, *J* = 5.7 Hz, 1 H), 7.78 – 7.74 (m, 2 H), 7.12 – 7.11 (m, 2 H), 7.11 – 7.10 (m, 1 H), 6.88 (dd, *J* = 7.9 Hz, *J* = 2.3 Hz, 1 H), 6.78 (bs, 1 H), 4.64 (d, *J* = 5.7 Hz, 2 H), 3.87 (s, 1 H).

^{13}C-NMR (126 MHz, acetone-d_6): δ [ppm] = 160.2, 154.9, 150.5, 131.0, 122.2, 115.1, 109.5, 104.9, 55.48, 38.29.

LC-MS: m/z: 350 $(M+H)^+$

Characterization of *N*-((1-(4-(trifluoromethoxy)phenyl)-1*H*-1,2,3-triazol-4-yl)methyl)-2-(trifluoromethyl)pyridin-4-amine (compound **12**): according to general procedure 5, 4-(trifluoromethoxy)aniline was used in the reaction and yielded 35.5 mg (88.0 µmol, 88%) of a colorless solid.

^{1}H-NMR (500 MHz, acetone-d_6): δ [ppm] = 8.61 (s, 1 H), 8.21 (d, *J* = 5.7 Hz, 1 H), 8.04 – 8.01 (m, 2 H), 7.58 – 7.56 (m, 2 H), 7.13 (d, *J* = 2.3 Hz, 1 H), 6.88 (dd, *J* = 7.9 Hz, *J* = 2.3 Hz, 1 H), 6.83 (bs, 1 H), 4.68 (d, *J* = 5.7 Hz, 2 H).

HRMS (ESI): $C_{16}H_{12}F_6N_5O^+$ $(M+H)^+$ calculated: 404.09406 found: 404.09394

Characterization of 2-(Trifluoromethyl)-*N*-((1-(4-(trifluoromethyl)phenyl)-1*H*-1,2,3-triazol-4-yl)methyl)pyridin-4-amine (compound **13**): the compound was prepared according to the general procedure 5. 4-(trifluoromethyl)aniline was used in the reaction and yielded 32.2 mg (83.2 µmol, 83%) of a colorless solid after purification *via* automated flash chromatography (PE/EtOAc).

LC-MS: m/z: 388 $(M+H)^+$

Characterization of 4-(4-(((2-(trifluoromethyl)pyridin-4-yl)amino)methyl)-1*H*-1,2,3-triazol-1-yl)benzonitrile (compound **14**): according to the general procedure 5, 4-aminobenzonitrile was used in the reaction and yielded 23.9 mg (69.4 µmol, 69%) of a colorless solid.

^{1}H-NMR (500 MHz, acetone-d_6): δ [ppm] = 8.72 (s, 1 H), 8.21 (d, *J* = 5.7 Hz, 1 H), 8.15 – 8.13 (m, 2 H), 8.03 – 8.01 (m, 2 H), 7.12 (d, *J* = 2.3 Hz, 1 H), 6.88 (dd, *J* = 7.9 Hz, *J* = 2.3 Hz, 1 H), 6.85 (bs, 1 H), 4.69 (d, *J* = 5.7 Hz, 2 H).

^{13}C-NMR (126 MHz, acetone-d_6): δ [ppm] = 154.9, 150.5, 146.5, 140.4, 134.4, 121.2, 120.9, 118.0, 109.5, 104.9, 38.18.

HRMS (ESI): $C_{16}H_{12}F_3N_6^+$ (M+H)$^+$ calculated: 345.10701 found: 345.10626

Characterization of *N*-((1-(4-phenoxyphenyl)-1*H*-1,2,3-triazol-4-yl)methyl)-2-(trifluoromethyl)pyridin-4-amine (compound **4**): according to the general procedure 5, 4-phenoxyaniline was used in the reaction and yielded 39.5 mg (96 µmol, 96%) of a grey solid.

^{1}H-NMR (300 MHz, DMSO-d_6): δ [ppm] = 8.72 (s, 1 H), 8.20 (d, *J* = 5.7 Hz, 1 H), 7.89 (m, 2 H), 7.64 (m, 1 H), 7.45 (m, 2 H), 7.21 (m, 3 H), 7.11 (m, 2 H), 7.06 (d, *J* = 2.2 Hz, 1 H), 6.83 (dd, *J* = 5.7 Hz, *J* = 2.2 Hz, 1 H), 4.53 (d, *J* = 5.7 Hz, 2 H).

^{13}C-NMR (75 MHz, DMSO-d_6): δ [ppm] = 156.9, 155.9, 154.3, 149.9, 145.1, 132.0, 130.2, 124.1, 122.1, 121.4, 119.3, 119.1, 37.26.

HRMS (ESI): $C_{21}H_{17}F_3N_5O^+$ (M+H)$^+$ calculated: 412.13797 found: 412.13705

Characterization of *N*-((1-(4-(phenylamino)phenyl)-1*H*-1,2,3-triazol-4-yl)methyl)-2-(trifluoromethyl)pyridin-4-amine (compound **15**): the compound was prepared according to the general procedure 5. *N*^{1}-Phenylbenzene-1,4-diamine (18.4 mg, 0.100 mmol) was used in the reaction and the title compound was afforded as an off-white solid (27.4 mg, 66.8 µmol, 67%) after purification *via* automated flash chromatography (PE/EtOAc).

HRMS (ESI): $C_{21}H_{17}F_3N_6^+$ (M+H)$^+$ calculated: 411.15396 found: 411.15317

Characterization of *N*-((1-(3,4-dichlorophenyl)-1*H*-1,2,3-triazol-4-yl)methyl)-2-(trifluoromethyl)pyridin-4-amine (compound **16**): according to the general procedure 5, 3,4-dichloroaniline and yielded 23.4 mg (60.0 µmol, 60%) of a colorless solid.

^{1}H-NMR (500 MHz, acetone-d_6): δ [ppm] = 8.67 (s, 1 H), 8.21 (d, *J* = 5.8 Hz, 1 H), 8.13 (d, *J* = 2.4 Hz, 1 H), 7.92 (dd, *J* = 8.9 Hz, *J* = 2.6 Hz, 1 H), 7.79 (d, *J* = 8.7 Hz, 1 H), 7.12 (d, *J* = 2.3 Hz, 1 H), 6.88 (d, *J* = 5.7 Hz, *J* = 2.3 Hz, 1 H), 6.84 (bs, 1 H), 4.68 (d, *J* = 5.8 Hz, 2 H).
^{13}C-NMR (75 MHz, DMSO-d_6): δ [ppm] = 154.9, 150.5, 137.0, 132.1, 122.2, 120.3, 109.5, 104.9, 38.20.
HRMS (ESI): $C_{15}H_{11}Cl_2F_3N_5^+$ $(M+H)^+$ calculated: 388.03381 found: 388.03388

Characterization of *N*-((1-(3-chloro-4-methoxyphenyl)-1*H*-1,2,3-triazol-4-yl)methyl)-2-(trifluoromethyl)pyridin-4-amine (compound **17**): according to general the procedure 5, 3-chloro-4-methoxyaniline was used in the reaction and yielded 28.0 mg (73.0 µmol, 73%) of a colorless solid.
^{1}H-NMR (500 MHz, acetone-d_6): δ [ppm] = 8.52 (s, 1 H), 8.22 (d, *J* = 5.6 Hz, 1 H), 7.90 (d, *J* = 2.6 Hz, 1 H), 7.80 (dd, *J* = 9.0 Hz, *J* = 2.8 Hz, 1 H), 7.31 (d, *J* = 8.9 Hz, 1 H), 7.12 (d, *J* = 2.3 Hz, 1 H), 6.88 (dd, *J* = 5.7 Hz, *J* = 2.2 Hz, 1 H), 6.81 (bs, 1 H), 4.65 (d, *J* = 5.6 Hz, 2 H), 3.98 (s, 3H).
^{13}C-NMR (75 MHz, DMSO-d_6): δ [ppm] = 155.6, 154.9, 150.5, 145.8, 131.1, 123.0, 122.4, 121.2, 120.4, 113.4, 109.5, 104.9, 56.44, 38.26.
HRMS (ESI): $C_{16}H_{14}ClF_3N_5O^+$ $(M+H)^+$ calculated: 384.08335 found: 384.08276

Characterization of *N*-((1-(3-chloro-4-(trifluoromethoxy)phenyl)-1*H*-1,2,3-triazol-4-yl)methyl)-2(trifluoromethyl) pyridin-4-amine (compound **18**): according to the general procedure 5, 3-chloro-4-(trifluoromethoxy)aniline was used in the reaction and yielded 30.6 mg (69.9 µmol, 70%) of a colorless solid.
^{1}H-NMR (500 MHz, acetone-d_6): δ [ppm] = 8.33 (d, *J* = 5.7 Hz, 1 H), 7.93 (m, 2 H), 7.69 (dd, *J* = 8.9 Hz, *J* = 2.6 Hz, 1 H), 7.51 (m, 1 H), 6.92 (d, *J* = 2.3 Hz, 1 H), 6.68 (dd, *J* = 5.7 Hz, *J* = 2.3 Hz, 1 H), 5.25 (m, 1 H), 4.63 (d, *J* = 5.6 Hz, 2 H).
^{13}C-NMR (75 MHz, DMSO-d_6): δ [ppm] = 153.5, 150.5, 149.2, 148.8, 145.3, 135.5, 130.0, 129.2, 123.7, 122.9, 122.0, 119.7, 118.63, 109.3, 107.8, 38.48.
HRMS (ESI): $C_{16}H_{11}ClF_6N_5O^+$ $(M+H)^+$ calculated: 438.05508 found: 438.05444

Characterization of *N*-((1-(3-chloro-4-phenoxyphenyl)-1*H*-1,2,3-triazol-4-yl)methyl)-2-(trifluoromethyl)pyridin-4-amine (compound **19**): the compound was prepared according to

the general procedure 5. 3-Chloro-4-phenoxyaniline (75 mg, 0.342 mmol) was used. The title compound was obtained as a colorless solid (43 mg, 0.337 mmol, > 98%).

^{1}H-NMR (500 MHz, acetone-d$_6$): δ [ppm] = 8.32 (d, *J* = 5.6 Hz, 1 H), 7.89 (s, 1 H), 7.85 (d, *J* = 2.6 Hz, 1 H), 7.54 (dd, *J* = 8.9 Hz, *J* = 2.6 Hz, 1 H), 7.39 (m, 2 H), 7.19 (m, 1 H), 7.05 (m, 3 H), 6.92 (d, *J* = 2.2 Hz, 1 H), 6.67 (d, *J* = 5.7 Hz, *J* = 2.3 Hz, 1 H), 5.34 – 5.31 (m, 1 H), 4.62 (d, *J* = 5.6 Hz, 2 H).

^{13}C-NMR (126 MHz, DMSO-d$_6$): δ [ppm] = 155.9, 153.7, 153.5, 150.3, 149.0, 148.6, 144.9, 132.4, 130.1, 126.5, 124.4, 123.5, 123.1, 120.3, 120.0, 119.9, 111.1, 109.2, 104.8, 38.43.

HRMS (ESI): $C_{21}H_{16}ClF_3N_5O^+$ $(M+H)^+$ calculated: 446.09899 found: 446.09830

Synthesis and characterization of 2-Chloro-*N*-((1-(4-phenoxyphenyl)-1*H*-1,2,3-triazol-4-yl)methyl)-6-(trifluoromethyl)pyridin-4-amine (**VI**):

To a solution of 4-phenoxyaniline (18.5 mg, 0.100 mmol) in MeCN (1 mL), *tert*-butyl nitrite (0.110 mmol, 12.6 mg, 14.5 µL) was added at 0 °C. After stirring for 5 min, trimethylsilylazide (0.110 mmol, 13.3 mg, 15.2 µL) was slowly added. The mixture was stirred for 1 h, and 2-chloro-*N*-(prop-2-yn-1-yl)-6-(trifluoromethyl)pyridin-4-amine (28.2 mg, 0.120 mmol) was added at r.t. in one portion. After addition of DIPEA (0.120 mmol, 15.5 mg, 20.9 µL), the reaction mixture was purged with argon, and $CuSO_4 \cdot 5\ H_2O$ (2.5 mg, 10 mol%) and Na-ascorbate (3.9 mg, 20 mol%) were added. The mixture was again purged with argon and stirred for 16 h at r.t. Saturated NH_4Cl solution was added to the reaction mixture, and stirred for 5 min. Then, EtOAc and saturated EDTA solution were added, and the mixture was stirred for 15 min until the aqueous phase showed a clear blue color. The phases were separated, and the aqueous phase was extracted with EtOAc three times. The combined organic layers were dried over $MgSO_4$, filtered and concentrated *in vacuo*. The crude product was purified *via* automated flash chromatography (PE/EtOAc) and afforded the title compound as a brownish solid (41.5 mg, 0.932 mmol, 93%).

^{1}H-NMR (300 MHz, $CDCl_3$): δ [ppm] = 7.90 (s, 1 H), 7.67 – 7.64 (m, 2 H), 7.42 – 7.38 (m, 2 H), 7.21 – 7.18 (m, 1 H), 7.15 – 7.12 (m 2 H), 7.08 – 7.06 (m, 2 H), 6.87 (d, *J* = 2.0 Hz, 1 H), 6.69 (d, *J* = 2.0 Hz, 1 H), 5.56 (bs, 1 H), 4.60 (s, 2 H).

^{13}C-NMR (76 MHz, $CDCl_3$): δ [ppm] = 158.3, 156.1, 155.3, 152.8, 148.5, 143.8, 131.7, 130.1, 124.3, 122.4, 120.0, 119.6, 119.2, 108.0, 104.7, 38.55.

HRMS (ESI): $C_{21}H_{16}ClF_3N_5O^+$ $(M+H)^+$ calculated: 446.09899 found: 446.09899

Synthesis and characterization of *N*[4]-((1-(4-phenoxyphenyl)-1H-1,2,3-triazol-4-yl)methyl)-6-(trifluoromethyl)pyridine-2,4-diamine (compound **20**):

A crimp vial was charged with 2-chloro-*N*-((1-(4-phenoxyphenyl)-1*H*-1,2,3-triazol-4-yl)methyl)-6-(trifluoromethyl)pyridin-4-amine **4.2** (45 mg, 0.100 mmol), *tert*-butyl carbamate (23 mg, 0.200 mmol) and Cs_2CO_3 (72 mg, 0.220 mmol). 1,4-dioxane (1.1 mL) was added, and the reaction mixture was purged with argon for 5 min. After addition of Pd-175 (3.9 mg, 5 mol%) the mixture was purged with argon for further 5 min, and the vial was heated at 90 °C. After stirring for 16 hours, the reaction mixture was filtered and subjected to automated flash chromatography using DCM/MeOH as an eluent which yielded the desired product as a beige solid (16.7 mg, 0.392 mmol, 39%).

^{1}H-NMR (500 MHz, acetone-d$_6$): δ [ppm] = 9.56 (bs, 1 H), 8.51 (s, 1 H), 7.89 – 7.86 (m, 2 H), 7.46 – 7.42 (m, 2 H), 7.22 – 7.20 (m, 1 H), 7.19 – 7.16 (m, 2 H), 7.11 – 7.09 (m, 2 H), 6.70 (d, *J* = 1.7 Hz, 1 H), 6.69 – 6.67 (m, 1 H), 6.03 (s, 1 H), 4.59 (d, *J* = 5.8 Hz, 2 H).

^{13}C-NMR (76 MHz, DMSO-d$_6$): δ [ppm] = 165.3, 157.9, 157.1, 156.9, 145.8, 133.0, 130.5, 124.5, 122.4, 121.1, 119.9, 100.7, 91.96, 38.45.

HRMS (ESI): $C_{21}H_{18}F_3N_6O^+$ $(M+H)^+$ calculated: 427.14887 found: 427.13721

2. Nanoparticle Preparation and Characterization

Squalenyl hydrogen sulfate was synthesized and characterized before use as previously described.[2] The drug-free and drug-loaded squalenyl hydrogen sulfate nanoparticles (SqNPs) were prepared using a single-step nanoprecipitation method as described in our previous study.[2]

Briefly, the drug-free SqNPs were prepared as follows: a solution of squalenyl hydrogen sulfate in tetrahydrofuran (THF) was prepared at a concentration of 10 mg/mL from which 0.1 mL was dropped slowly into 1 mL of MilliQ water under constant stirring allowing nanoprecipitation. Following the 30 min stabilization, organic solvent was evaporated, and a dispersion of SqNPs 1 mg/mL in aqueous medium was obtained. The concentration of SqNPs in aqueous solution can be varied by changing the initial concentration of squalenyl hydrogen sulfate in THF.

The preparation of drug-loaded SqNPs is as below.

Single drug-loaded SqNPs preparation: the hydrophobic compound (**4**) loaded SqNPs were prepared using nanoprecipitation method. The optimal compound (**4**):squalenyl hydrogen sulfate ratio was 0.8:9 (w/w) was solubilized in THF allowing the maximum encapsulation

efficiency (EE%) and loading capacity (LC%) of compound (**4**) in SqNPs. The positively charged tobramycin (Tob) was prepared in the aqueous solution, which was loaded on SqNPs surface *via* electrostatic interaction during the precipitating procedure. The optimal Tob/ squalenyl hydrogen sulfate ratio was 3:7 (w/w), providing the maximum EE% and LC% of Tob as well as allowing the long-term drug-loaded SqNPs stability.

The Tob and compound (**4**) co-loaded SqNPs were produced by simultaneously co-assembling squalenyl hydrogen sulfate with Tob and QSI **4**, using the above protocol. The Tob concentration (from 6.25 – 200 µg/mL) loaded in SqNPs was tuned by varying the Tob initial concentration in the aqueous phase.

The nanoparticle characteristics (hydrodynamic size, PDI, and ζ-potential) were determined using Dynamic Light Scattering (DLS, Zetasizer Nano-ZS, Malvern Instruments, Worcestershire, U.K.) equipped with a 4 mW He−Ne laser employing a wavelength of 633 nm and a backscattering angle of 173° at 25 °C. The reported size represents the z-average hydrodynamic diameter (intensity based) of three measurements.

The drug loading quantification was as follows: The loaded amount of Tob and compound (**4**) in SqNPs were determined indirectly by quantifying of the unloaded amount in the supernatant (in short, loaded amount = initial amount – amount in the supernatant). The quantification of Tob and compound **4** were done using liquid chromatography with tandem mass spectrometry (LC-MS/MS) methods developed in our laboratory. The system was operated by the standard software Xcalibur. A reverse phase C18 Accucore RP-MS (150·2.1 mm) column (Thermo Scientific, Waltham, MA, USA) was used as stationary phase to quantify Tob at 30 ^{o}C. The mobile phase was composed of solvent A (water containing 0.1% v/v formic acid, FA) and solvent B (acetonitrile containing 0.1% v/v trifluoroacetic acid, TFA), solvent ratio A:B was 95:5, and the flow rate was 300 µL/min, controlled by an Accela 1250 Pump. A standard curve was established at Tob concentrations ranged from 2.5 – 80 µg/mL in PBS. The quantification of QSI (**4**) was done as follows: Samples were diluted 1:100 in acetonitrile containing 500 nM internal standard (diphenhydramine). A calibration curve was created from 0.1 to 50 nM compound (**4**) by drying 20 µL of 10x concentrated solutions in ethyl acetate, followed by addition of 200 µL acetonitrile containing 500 nM internal standard. LC-MS/MS analysis was performed on an Ultimate 3000 system (degasser, pump, autosampler, column compartment) equipped with a Nucleodur C18 Pyramid column (150·2 mm, 5 µm, Macherey-Nagel, Düren, Germany) coupled to a TSQ Quantum Access MAX (Thermo Fisher Scientific, Waltham MA) with the following gradient conditions: A –

water (0.1% formic acid); B – acetonitrile (0.1% formic acid); flow 0.600 mL/min; 90% A for 1.0 min; 90 – 5% A in 0.7 min; 5% A for 1.8 min; equilibration at 90% A for 1.0 min. MS was operated in positive SRM mode with the following mass transitions: diphenhydramin (IS): 256.04 – 164.90; 256.04 – 166.90; QSI (**4**): 412.04 – 221.972. The EE% and LC% were calculated according to equations (S1) and (S2).

Equations:

$$\text{EE\%} = \frac{Weight\ of\ encapsulated\ drug\ in\ NPs}{Initial\ weight\ of\ used\ drug} \text{ x } 100 \qquad (S1)$$

$$\text{LC\%} = \frac{Weight\ of\ \ drug\ in\ NPs}{Weight\ of\ NPs} \text{ x } 100 \qquad (S2)$$

"Weight of nanoparticles (NPs)" was calculated as: Weight of nanoparticles = Weight of squalenyl hydrogen sulfate + Weight of encapsulated drug in nanoparticles.

The nanoparticle morphology was observed by cryo-TEM. For cryo-TEM investigations, samples were prepared using a Gatan (Pleasanton, OR, US) CP3 cryo plunger. Shortly, 3 µL of the solution were placed on a Plano (Wetzlar, Germany) S147-4 holey carbon grid, plotted for 2 s and plunged into undercooled liquid ethane at T = –165 °C. The grid was transferred under liquid nitrogen to a Gatan model 914 cryo-TEM sample holder operating at T = –170 °C. TEM bright field images were acquired using a JEOL (Akishima, Tokio, Japan) JEM-2100 LaB_6 equipped with a Gatan Orius SC1000 CCD camera under low-dose conditions.

The elemental composition of the drug-loaded SqNPs were investigated using TEM. The investigations were performed on a JEOL (Akishima, Tokio, Japan) JEM-2100 LaB_6 microscope equipped with a Thermo (Thermo Fisher Scientific, Schwerte, Germany) Noran NSS7 X-ray spectrometer and a Gatan (Pleasanton, Ca, United States) Orius SC1000A CCD camera. A drop of 10 µL nanoparticles dispersion was placed on a holey carbon grid (Plano, Wetzlar, Germany) and dried at ambient conditions. The energy dispersive X-Ray (EDX) spectra were acquired under a tilting angle of 25° for 60 seconds.

The *in vitro* release studies of Tob and compound **4** from the drugs co-loaded SqNPs were performed in PBS (pH 7.4) at 37 °C over the 24 h period. Briefly, Tob and QSI **4** co-loaded SqNPs having maximum LC% (~30% Tob and ~8% compound **4**) were dispersed in PBS to provide a final NP concentration of 10% (w/w). The whole system was then placed on a shaker at 400 rpm and 37 °C. The concentration of the released drugs was analyzed by collecting samples from the supernatant at the designated time points over 24 h. Compound **4** was extracted from the supernatant using ethyl acetate, which was then dried and re-dissolved in methanol prior to LC-MS/MS analysis. The receptor volume was kept constant by refilling

with an identical volume of PBS at each time point. The cumulative released drug percentage over 24 h was measured. Three independent experiments were conducted in triplicate, and results are expressed as the mean ± SD. The release profiles of Tob and compound **4** from the drugs co-loaded SqNPs are shown in Figure S1.

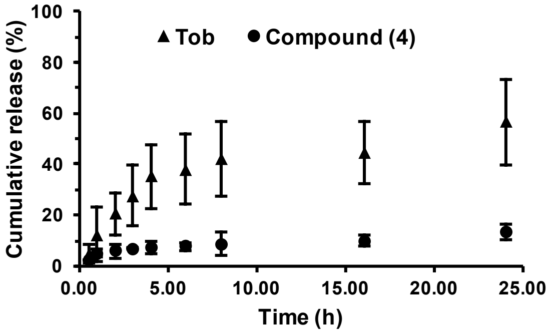

Figure S1. *Cumulative release profiles of Tob and compound (**4**) from the drugs co-loaded SqNPs. Three independent experiments were conducted in triplicate.*

3. Primary Activity Assays

3.1. Reporter Gene Assay

The inverse agonistic/antagonistic activity of potential PqsR ligands was evaluated in a β-galactosidase reporter gene assay in *E. coli* as previously described.[3] In short, test compounds were co-incubated with E. coli DH5α cells containing the plasmid pEAL08-2,[4] which expresses PqsR and the β-galactosidase LacZ under the control of the pqsA promoter. The inverse agonistic/antagonistic activity of compounds was measured in the presence of 50 nM PQS. After incubation, galactosidase activity was quantified photometrically and expressed as ratio of controls. The given IC50 values represent the mean of at least two independent experiments using at least eight different concentrations of test compound with n = 4. Non-linear regression analysis was performed using OriginPro 2020.

3.2. Pyocyanin assay

The pyocyanin inhibitory effect of the synthesized compounds and the compound (**4**) loaded SqNPs on PA14 wt were investigated by pyocyanin assay as described in previous work.[2,5] Briefly, PA14 wt was grown in PPGAS medium, overnight, at 37 °C, shaking at 200 rpm. The bacterial suspension was then centrifuged at 7.450 *g*, washed twice and diluted to a final OD600 (absorption at 600 nm) of 0.02 ($2{\cdot}10^7$ CFU/mL) in fresh PPGAS. 1.5 mL of the

culture was distributed in each well of the 24 well-plate. The free synthesized QSI prepared in DMSO, compound (**4**) loaded SqNPs were supplemented to bacteria-containing wells (1:100 dilutions). PA14 wt treated with either PBS or drug-free SqNPs (200 µg/mL) served as controls. Following 16 h incubation under aerobic conditions, pyocyanin in 900 µL of bacteria culture was extracted with chloroform and re-extracted with 0.2 M HCl. Pyocyanin production level was measured as the absorption at 520 nm, which was normalized to cell growth measured as OD600. 0.2 M HCl served as blank samples. Three independent experiments were conducted in triplicate. Non-linear regression analysis was performed using OriginPro 2020. Figure S2 shows the dose-response pyocyanin inhibitory assay of compound (**4**) loaded SqNPs on PA14 wt. The pyocyanin inhibitory efficacy was improved by a factor of two when using compound (**4**) loaded SqNPs (IC_{50} is 100 ± 0.018 nM) compared to free compound (**4**) prepared in DMSO (IC_{50} is 199 nM). It is noted that as reported in our prior study, the drug-free SqNPs did not show any effects on PA14 pyocyanin production.[2]

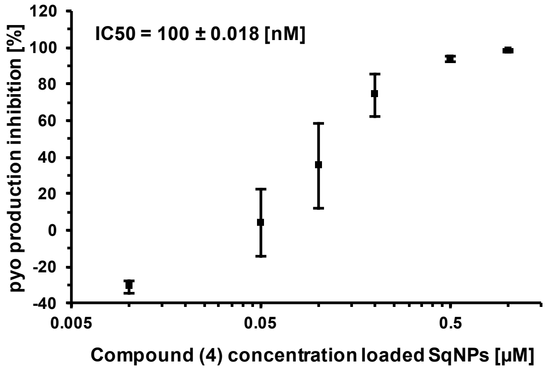

Figure S2. *The production of pyocyanin levels compared to control PA14 wide type (wt) of the samples treated with 200 µg/mL drug free SqNPs. Three independent experiments were conducted in triplicate.*

4. Secondary Activity Assays

4.1. Alkylquinolone Inhibition Assay

The inhibitory effect of the synthesized compounds on alkylquinolone production in PA was measured as previously described with the modification that wt PA14 was used instead of PA14*pqsH*.[6] Briefly, *P. aeruginosa* PA14 cultures were grown in the presence of DMSO as a control or DMSO solutions of inhibitors for 17 h. HHQ and PQS as well as HQNO were quantified from ethyl acetate extracts using 5,6,7,8-tetradeutero-2-heptyl-4(1H)-quinolone (HHQ-d4) as internal standard (IS). LC-ESI-MS/MS analysis was performed on a Dionex Ultimate 3000 HPLC system coupled to a TSQ Quantum Access MAX (Thermo Fisher

Scientific, Waltham MA) with the following conditions: Zorbax Eclipse XDB 80 Å C18 5 µm 4.6 x 50 mm column (Agilent, Santa Clara, CA, USA); eluent A – H_2O with 0.1% trifluoroacetic acid (TFA), 0.1% pentafluoropropionic acid (PFPA), 0.1% heptafluorobutyric acid (HFBA); eluent B – acetonitrile with 0.1% TFA, 0.1% PFPA, 0.1% HFBA; isocratic 50% A; flow – 0.7 mL/min. TSQ Quantum Access Max (Thermo Fisher Scientific, Waltham, MA, USA) instrument parameters: Spray voltage 3500 V, vaporizer temperature 370 °C, sheath gas pressure 35 psi, aux gas pressure 30 psi, capillary temperature 270 °C, collision pressure 1.5 mTorr, positive ionization mode. For AQ analyte reaction monitoring parameters see Table S1. Data acquisition and quantification was performed using Xcalibur software with the use of a calibration curve relative to the area of the IS. IC_{50} values were calculated according to the procedure of the pyocyanin assay (see above).

Table S1: LC-ESI-MS/MS reaction monitoring parameters of AQ analytes in positive ionization mode.

	**precursor ion m/z**	**product ion m/z**	**collision energy (V)**	**tube lens offset (V)**	**retention time (min)**
PQS	260.048	145.958	44	110	2.4
		174.927	30	110	
HHQ	244.050	158.944	31	100	2.3
		171.943	33	100	
HQNO	260.036	158.908	28	110	3.0

4.2. MIC Assay

Chemical and biological materials for *in vitro* assays: All chemicals were purchased from Sigma-Aldrich unless otherwise specified. Yeast extract was obtained from Fluka. Bacto™ Tryptone were obtained from BD Biosciences. Luria Bertani (LB) agar was obtained from Carl Roth. Gibco® PBS was obtained from Life Technologies. Purified water was prepared by a Milli-Q water purification system (Merk Millipore, Billerica, MA). The PA strain PA14 wild type (PA14 wt) was purchased from DSMZ, the German Collection of Microorganism and Cell Cultures GmbH (PA14 = DSMZ19882) and cultured in minimal proteose peptone glucose ammonium salt (PPGAS) medium. PPGAS was composed of 1 g/L NH_4Cl, 1.5 g/L KCl, 19 g/L Tris-HCl, 10 g/L peptone, 5 g/L glucose and 0.1 g/L $MgSO_4{\cdot}7H_2O$. The pH value of the medium was adjusted to 7.2 ± 0.2 and sterilized before use. Agar solution was

composed of 15.5 g/L LB agar, 10 g/L peptone, 5 g/L NaCl and 5 g/L yeast extract, the resulting solution was sterilized before being plated.

The minimum inhibitory concentration (IC) of drug-free SqNPs, compound **4** prepared either in dimethyl sulfoxide (DMSO) or loaded in SqNPs, free Tob, Tob-loaded SqNPs, and Tob and compound **4** co-loaded SqNPs against planktonic PA14 wt were determined by standard microbroth dilution assays in 96-well plates. PA14 wt suspension was prepared from mid-log cultures in PPGAS medium and diluted to OD600 (absorption at 600 nm) 0.02 ($2 \cdot 10^7$ CFU/mL (CFU, colony-forming units)). PBS served as a control. Following 16 h incubation at 37 °C, IC_{90} values were determined by sigmoidal curve fitting of absorption values (600 nm) that were measured using a Tecan microplate reader Infinite M200Pro (Tecan, Crailsheim, Germany). The IC_{90} values are concentrations at which the growth of bacteria is inhibited by 90%. Three independent experiments were conducted in triplicate. Results are summarized in Table S2.

Table S2. *MIC assay against PA14 wt. Three independent experiments were conducted in triplicate.*

**Samples**	**MIC (IC_{90} value) against PA14 wt**
Drug-free SqNPs	> 200 µg/mL
Compound (**4**) prepared in DMSO	> 20 µM
Compound (**4**)-loaded SqNPs	> 20 µM [(a)]
Free Tob	3.125 – 6.25 µg/mL
Tob loaded SqNPs	3.125 – 6.25 µg/mL [(b)]
Tob and compound (4) co-loaded SqNPs ((**4**) concentration 20 µM)	3.125 – 6.25 µg/mL [(b)]

[(a)] Compound (**4**) concentration
[(b)] Tob concentration

4.3. Minimum Biofilm Eradicating Concentration (MBEC) assay

Chemical and biological materials for *in vitro* assays. All chemicals were purchased from Sigma-Aldrich unless otherwise specified. Yeast extract was obtained from Fluka. Bacto™ Tryptone were obtained from BD Biosciences. Luria Bertani (LB) agar was obtained from Carl Roth. Gibco® PBS was obtained from Life Technologies. Purified water was prepared by a Milli-Q water purification system (Merk Millipore, Billerica, MA). The PA strain PA14

wild type (PA14 wt) was purchased from DSMZ, the German Collection of Microorganism and Cell Cultures GmbH (PA14 = DSMZ19882) and cultured in minimal proteose peptone glucose ammonium salt (PPGAS) medium. PPGAS was composed of 1 g/L NH_4Cl, 1.5 g/L KCl, 19 g/L Tris-HCl, 10 g/L peptone, 5 g/L glucose and 0.1 g/L $MgSO_4 \cdot 7H_2O$. The pH value of the medium was adjusted to 7.2 ± 0.2 and sterilized before use. Agar solution was composed of 15.5 g/L LB agar, 10 g/L peptone, 5 g/L NaCl and 5 g/L yeast extract, the resulting solution was sterilized before being plated. The minimum inhibitory concentration (IC) of drug-free SqNPs, compound **4** prepared either in dimethyl sulfoxide (DMSO) or loaded in SqNPs, free Tob, Tob-loaded SqNPs, and Tob and compound **4** co-loaded SqNPs against planktonic PA14 wt were determined by standard microbroth dilution assays in 96-well plates. PA14 wt suspension was prepared from mid-log cultures in PPGAS medium and diluted to OD600 (absorption at 600 nm) 0.02 ($2 \cdot 10^7$ CFU/mL (CFU, colony-forming units)). PBS served as a control. Following 16 h incubation at 37 °C, IC_{90} values were determined by sigmoidal curve fitting of absorption values (600 nm) that were measured using a Tecan microplate reader Infinite M200Pro (Tecan, Crailsheim, Germany). The IC_{90} values are concentrations at which the growth of bacteria is inhibited by 90%. Three independent experiments were conducted in triplicate. Results are summarized in Table S2.

The MBECs of free Tob, Tob-loaded SqNPs, free Tob plus free compound **4** prepared in DMSO, and Tob and compound (**4**) co-loaded SqNPs were determined by the MBEC assay as reported in previous study.[2,7] Briefly, PA14 wt was grown in PPGAS medium, overnight at 37 °C shaking at 200 rpm. The bacterial suspension was then centrifuged at 7,450 *g*, washed twice and diluted to a final OD600 of 3.0 (10^{15} CFU/mL) in fresh PPGAS. 0.2 mL of the bacterial culture was distributed in each well of the 96-well plate. The biofilm in bacteria-containing plates were formed under aerobic conditions (37 °C, without shaking) for 24 h. Following a removal of planktonic bacteria, the 24 h-old biofilms were washed twice with PBS and fed with fresh PPGAS medium. Stock solution of free Tob, Tob-loaded SqNPs, free Tob plus free compound **4**, or Tob and compound **4** co-loaded SqNPs was diluted (1:100 dilution) to bacterial biofilm-containing wells to obtain the designated final concentrations. Biofilm treated with PBS, drug-free SqNPs, free compound **4** and compound **4**-loaded SqNPs served as controls. After being further incubated for 24 h at 37 °C without shaking, all samples were washed twice with PBS, fed with 200 mL of fresh PPGAS and sonicated for 10 min to disperse the biofilms. The biofilm-eradicating efficacy was assessed according to viable bacterial load determined using dilution in PBS/0.05% Tween20 and plating on agar

plates to count CFU after overnight incubation at 20 °C. The concentration of compound **4** was kept constant at 20 µM in all experiments. Three independent experiments were conducted in triplicate. Figure S3 shows that the drug-free SqNPs and 20 µM of compound **4** either in free form or loaded in SqNPs did not show any PA14 wt biofilm inhibitory effects. Figure S4 shows the results of Figure 5 in the manuscript as curves to visualize the difference in biofilm eradication potential. Using GraphPad Prism 8.4.2, the AUC was calculated for each condition. One-way ANOVA (Tukey's multiple comparisons test) of the calculated AUCs demonstrated the following p values in comparison to the Free Tob group: 0.2185 (Tob-loaded SqNPs, ns), 0.0035 (Tob & **4**, **), <0.0001 (Tob & **4** co-loaded SqNPs).

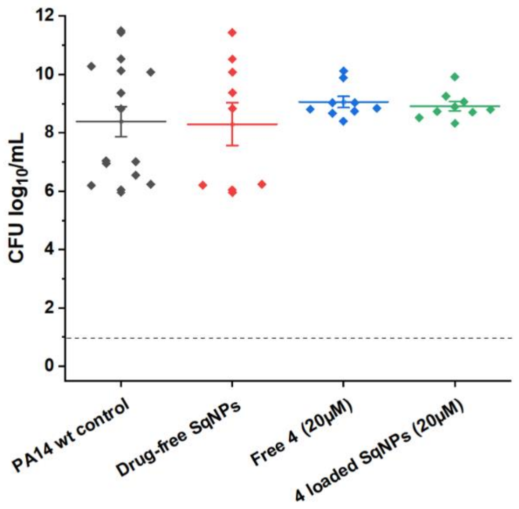

Figure S3. *Minimum biofilm eradicating concentration (MBEC) assay on PA14 wt biofilm grown in PPGAS medium for 24h: biofilms were treated with either PBS, drug-free SqNPs, free compound (**4**) or compound (**4**) loaded SqNPs. These samples served as controls. The concentration of compound (**4**) was 20 µM and kept constant in all assays using compound (**4**). After a 24h treatment, efficacy was assessed by determination of colony-forming units per millilitre (CFU/mL). CFU/mL values are depicted logarithmically for n = 16. The dotted line indicates the detection limit.*

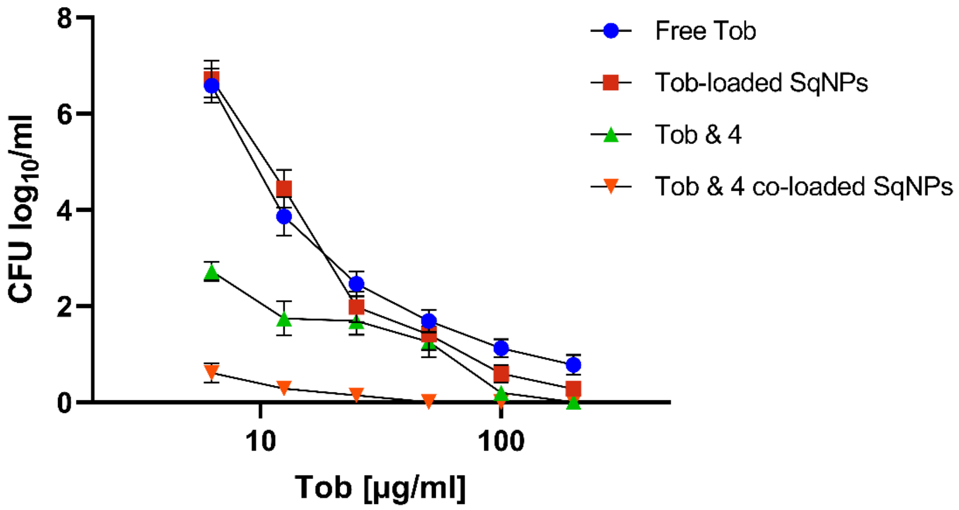

Figure S4. *Combined depiction of data shown in Figure 5 from the main text comparing the concentration-dependent biofilm eradication by free Tob (blue spheres), Tob-loaded SqNPs (red squares), Tob in combination with* ***4*** *(green triangles) and Tob +* ***4*** *co-loaded SqNPs (orange triangles).*

5. *In vitro* ADME/T and Safety Pharmacology

5.1. CEREP off-targets assay

Experiments towards possible off-target effects were conducted following Eurofins Discovery protocol. Detailed information about the assays can be found in:

https://www.eurofinsdiscoveryservices.com/catalogmanagement/viewitem/SafetyScreen44-Panel-Cerep/P270

Table S3. *Possible CEREP off-targets.*

entry	off-target	QSI **4** (%binding @10µM)	QSI **17** (%binding @10µM)	Assay method
1	β_2 receptor	18.03	85.3	Antagonist radioligand
2	5-HT_{2B}	55.25	73.79	Agonist radioligand
3	5-HT_{2A}	65.15	22.02	Agonist radioligand

4	AR	-10.10	57.04	Agonist radioligand
5	α_{1A} receptor	40.59	55.42	Antagonist radioligand
6	Dopamine transporter	97.25	28.91	Antagonist radioligand
7	Norepinephrine transporter	82.52	8.58	Antagonist radioligand
8	Ca^{2+} channel (L, dihydropyridine site)	78.29	30.36	Antagonist radioligand
9	Na^{+} channel (site 2)	54.62	-27.90	Antagonist radioligand

5.2. CYP and hERG inhibition

Assessment of *in vitro* drug safety regarding Cytochrom P450 (CYP) inhibition and inhibiton of human ether-à-go-go related gene (hERG) channel with a functional (patch-clamp-based) assay were performed at Cyprotex. Detailed information about the assays can be found in: https://www.cyprotex.com/admepk/in-vitro-metabolism/cytochrome-p450-inhibition, and https://www.cyprotex.com/toxicology/cardiotoxicity/hergsafety

5.3. Cytotoxicity Assays

Hep G2 or HEK293 cells (2×10^5 cells per well) were seeded in 24-well, flat-bottomed plates. Culturing of cells, incubations, and OD measurements were performed as described previously with slight modifications.[8] Twenty-four hours after seeding the cells, the incubation was started by the addition of test compound in a final DMSO concentration of 1%. The living cell mass was determined after 48 h. At least two independent measurements were performed for each compound.

5.4. Kinetic Turbidimetric Solubility Assay

The desired compounds were sequentially diluted in DMSO in a 96-well plate. A 3 µL of each well were transferred into another three 96-well plates, followed by addition of 147 µL of PBS. Plates were shaken for 5 min at 600 rpm at room temperature (r.t.), and the absorbance at 620 nm was measured. Absorbance values were normalized by blank

subtraction and plotted using GraphPad Prism 8.4.2 (GraphPad Software, San Diego, CA, USA). Solubility (S) was determined based on the First X value of AUC function using a threshold of 0.005.

5.5. Metabolic stability

For the evaluation of combined phase I and phase II metabolic stability, the compound (1 µM) was incubated with 1 mg/mL pooled liver S9 fraction from the respective species (Xenotech), 2 mM NADPH, 1 mM UDPGA, 10 mM $MgCl_2$, 5 mM GSH and 0.1 mM PAPS at 37 °C for 0, 5, 15, 30 or 60 min. The metabolic stability of Testosterone (1 µM) and verapamil (1 µM) were determined in parallel to confirm the enzymatic activity of the S9 fraction. The incubation was stopped by precipitation of S9 enzymes with 2 volumes of cold acetonitrile containing internal standard (750 nM Diphenhydramine). Samples were stored on ice for 10 min and precipitated protein was removed by centrifugation (10 min, 4 °C, 17,000 g). Concentration of the remaining test compound at the different time points was analyzed by LC-MS/MS (TSQ Quantum Access MAX, Thermo Fisher, Dreieich, Germany) and used to determine half-life ($t_{1/2}$). Stability of compound **4** in further S9 fractions are summarized in Table S4.

Table S4. *Metabolic stability studies in various S9 fractions.*

species	$t_{1/2}$ (QSI **4**) [min]
human liver S9	67.6 ± 9.0
mouse liver S9	19.9 ± 7.5
rat liver S9	24.2 (n=1)
rabbit liver S9	20 ± 10
guinea pig liver S9	92.5 ± 5.4
dog liver S9	72.2 ± 7.6
monkey liver S9	22.8 ± 6.7

5.6. Permeability studies

Permeability of compound **4** and **17** was assessed *in vitro* with Calu-3 HTB-55 cell line (ATCC). Cells were cultivated in Minimum Essential Medium supplemented with Earle´s salts, L-Glutamine, 10% FCS, 1% non-essential amino acids (NEAA) and 1mM Sodium

Pyruvate. Passages between 35 and 55 were used, medium was changed every 2-3 days. For experiments, cells were harvested using Trypsin/EDTA and seeded on Transwell® inserts 3460 at a density of $1 \cdot 10^5$ cells. Cells were grown at air-liquid interface beginning day 3 and used for Transport studies on day 11-13. TEER values exceeded 300 $\Omega \cdot cm^2$ before beginning Transport studies. For experiments, Krebs-Ringer solution with 1% BSA was used and cells were accommodated to the buffer for at least 1h with no decrease in TEER. 200 µL samples were taken in regular intervals from the apical side (time intervals 0, 20, 40, 60, 90, 120, 180, 240 minutes) and replenished with fresh buffer. TEER was monitored during the experiment and epithelial barriers were considered compromised if the TEER fell below 300 during 4 h of experiment duration. Sodium-fluorescein and Ciprofloxacin·HCl were used as a control. 50 µL of sample was mixed with 150 µL of ice-cold acetonitrile containing internal standard diphenhydramine (1 µM) and the concentration of compound was analyzed with LC-MS/MS.

6. *In vivo* Experiments

6.1. Tolerability of QSI (**4**) and SqNP-assembly in Mice

Two tolerability studies were performed: A) assessing the maximum tolerated dose (MTD) in mice after i.v. application and B) *in vivo* tolerability QSI-loaded SqNP after intracheal administration.

A) Mouse MTD of QSI **4**.

Acute single dose toxicity study in the mouse was performed by the CRO Aurigon (ATRC Aurigon Toxicological Research Center Ltd, Hungary). Procedures and facilities complied with the requirements of the Directive 2010/63/EU from September 22, 2010 and subsequent amendments on the approximation of laws, regulations and administrative provisions of the Member States regarding the protection of animals used for experimental and other scientific purposes. The necessary permission of the Institutional Animal Care and Use Committee (IACUC) at ATRC were obtained prior to commencing the study. The institution ATRC is registered at the Authority as an experimental animal usage facility and has an authorization for animal use.

This study was conducted in compliance with the principles of Hungarian Act 1998:

XXVIII regulating animal protection (latest modified by Act 2011 CLVIII) and in Government Decree 40/2013 on animal experiments.

Handling and care of the animals will be conducted according to the Guide for the Care and Use of Laboratory Animals, NRC, 2011.

Compound **4** solution was prepared as follows. Stock solutions of 10x the desired administered concentrations were prepared in DMSO. These stocks can be stored at -30 – -15 °C for at least 6 months. For application, stock solutions in DMSO were to be diluted by 1:10 with a mixture of PEG400 and sodium chloride aqueous solution 0.9% (six volumes PEG400 to four volumes NaCl-solution). Final vehicle concentration was 54% PEG400, 36% NaCl solution (aqueous, 0.9%), 10% DMSO. No test item related clinical symptoms were reported to be observed after 24 h up to a test dosing of 60 mg/kg.

B) Tolerability QSI-loaded SqNP after intracheal administration.

The *in vivo* tolerability study was performed on ten-week old female C57BL/6N mice. Mice were anesthetized and administered intra-tracheally (1 µL/g) a 1 mg/kg equivalent dose of compound (**4**) which was either formulated by loading in SqNPs or dispersing in a mixture solution of methylcellulose (0.5%), Tween-80 (0.2%) in 0.9% NaCl (called Meth-Cell). Mice dosed with 0.9% NaCl served as controls. Mice behavior and body weight were monitored over 24 h period. At 24 h post-injection, the mice were euthanized, lungs were collected, and the blood leukocytes as well as the absolute leukocytes (including segented, lymphocytes, monocytes and eosinophiles) were counted to access the tolerability. Five mice were included in each group. Generally, the mice appeared normal and active in all groups. Figure S4 shows that mice dosed with 1 mg/kg equivalent compound (**4**) loaded SqNPs did not cause significant weight loss after 24 h. The same holds true for the lungs mass. Moreover, the loading compound (**4**) into SqNPs did not result in the trigger of both local and systemic immune response. In conclusion, the compound (**4**) loaded SqNPs showed good *in vivo* compatibility in mouse.

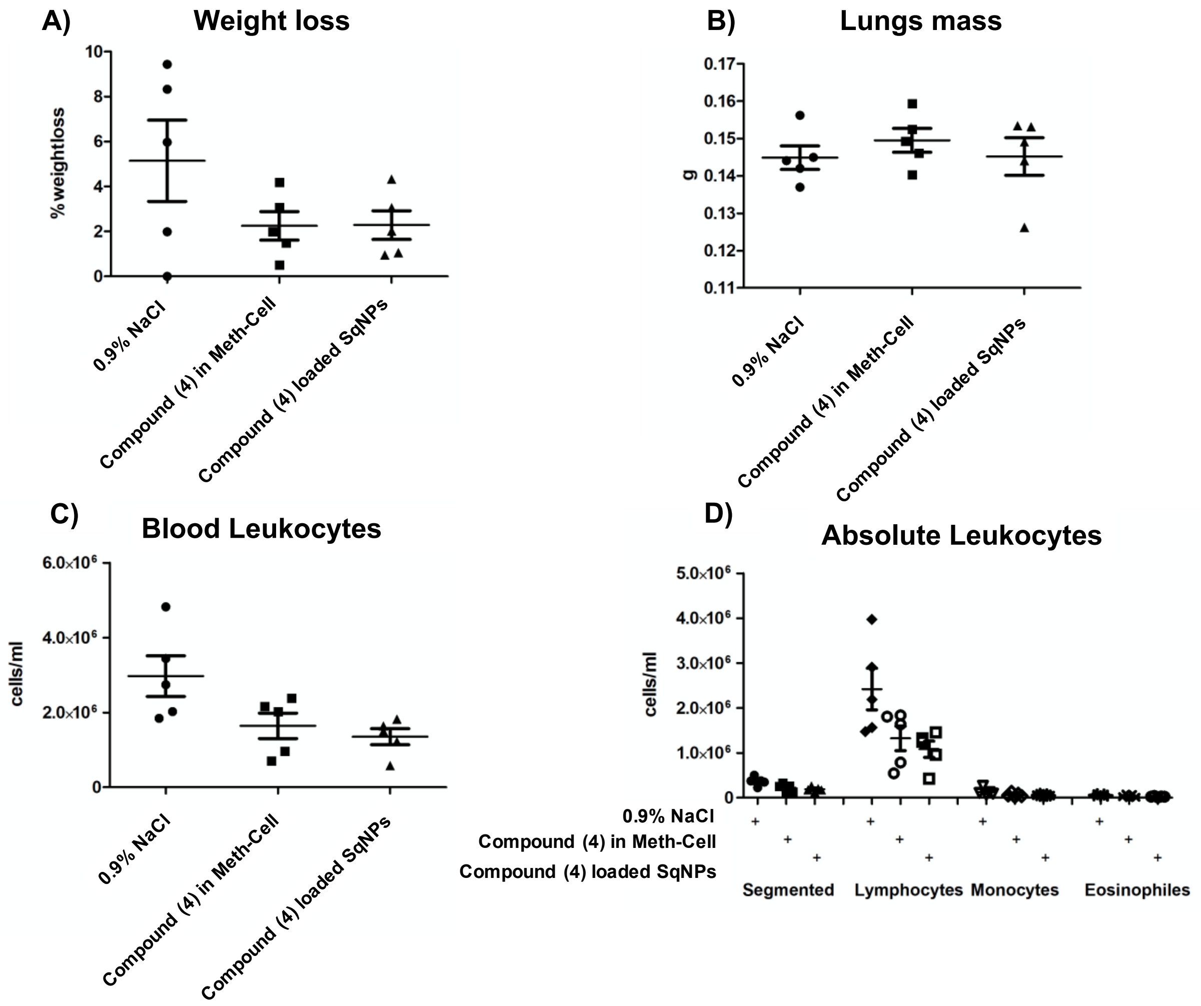

*Figure S5. In vivo tolerability of compound **4** in a mouse model. Mice were administered intra-tracheally (1 μL/g) a 1 mg/kg equivalent dose of compound **4** which was either formulated by loading in SqNPs or dispersing in a mixture solution of methylcellulose (0.5%), Tween-80 (0.2%) in 0.9% NaCl (called Meth-Cell). Mice dosed with 0.9% NaCl served as controls. In vivo tolerability was accessed by (A) weight loss percentage; (B) lungs mass; (C) blood leukocytes; and (D) absolute leukocytes. Five mice were included in each group.*

6.2. PK Sample Preparation and Analysis

Groups of male CD1 mice (25-30 g, Charles River UK) were housed in groups of up to 6 and maintained under a 12 h light/dark cycle with free access to food and water. Temperature and humidity were controlled according to U.K. HO regulations. On the day of the study, mice were briefly anaesthetized with isoflurane before suspending the mouse by its upper incisors on a board held at an angle of approx. 45 degrees. Mice were then dosed intratracheally with the compound (**4** or Tob) at the designated does formulated in ethanol/tyloxapol/glycerol/phosphate-buffered saline (PBS) [10:1:5:84] in a 1 mL/kg dosing volume up to a maximum of 30 μL per mouse. A laryngoscope was used to facilitate dosing. Animals were allowed to recover before replacing into their home cages. Terminal blood

samples were then taken from groups of 3 mice at each of eight time points post-dose by cardiac puncture under CO_2. Blood samples were placed in tubes on ice containing EDTA anticoagulant. After gentle mixing, samples were centrifuged (10,000 rpm x 3 min) and two 50 µL aliquots taken into fresh tubes that were immediately frozen on dry ice. Following blood sample collection, tracheas were exposed and a small incision made. An 18 Gauge plastic tube was then inserted through the incision into the trachea and tied in place. Aliquots of 0.8 mL of sterile saline were then infused into and then withdrawn from the lungs and the collected bronchoalveolar lavage (BAL) sample placed into a tube. This process was repeated two more times, and the samples from each mouse pooled. The resulting samples were weighed and mixed, before a 1 mL aliquot was taken for analysis and frozen on dry ice. Immediately after collecting the final BALF sample, lung samples were excised and excess tissue removed, before rinsing in saline, blotting and weighing. Samples were then snap frozen by immersion in liquid nitrogen. All samples were stored at -20 °C until sent for analysis on dry ice.

All PK plasma samples were analyzed *via* HPLC-MS/MS using an Agilent 1290 Infinity II HPLC system coupled to an AB Sciex QTrap 6500plus mass spectrometer. First, a calibration curve was prepared by spiking different concentrations of compound **4** or Tob (Fa. Sigma) into plasma, homogenized lung from CD-1 mice or 0.9% sodium chloride solution as matrix for broncheoalveolar lavage fluid (BALF). The lower limits of quantification are indicated in Table S5. Caffeine was used as internal standard. In addition, quality control samples (QCs) were prepared for **4** and Tob in plasma, homogenized lung and 0.9% sodium chloride solution/BALF.

The following extraction procedure was used for **4**: plasma: 7.5 µL of a plasma sample (calibration samples, QCs or PK samples) was extracted with 37.5 µL of acetonitrile containing 12.5 ng/mL caffeine as internal standard for 10 min at 2000 rpm on an Eppendorf MixMate® vortex mixer. Lung: homogenized samples were diluted to 50 mg/mL lung tissue in 0.9% sodium chloride solution. A 50 µL of a lung sample (calibration samples, QCs or PK samples) was extracted with 15 µL water containing 10% formic acid and 50 µL of acetonitrile containing 12.5 ng/mL caffeine as internal standard for 10 min at 2.000 rpm on an Eppendorf MixMate® vortex mixer. BALF: 200 µL of methanol were added to 100 µL of a BALF sample (calibration samples, QCs or PK samples). Samples were concentrated in an Eppendorf concentrator until dryness at 30 °C. A 30 µL of water containing 10% formic acid

and 50 µL of acetonitrile containing 12.5 ng/mL caffeine were added to the dried samples and dissolved for 10 min at 2,000 rpm on an Eppendorf MixMate® vortex mixer.

The following extraction procedure was used for Tob: A 7.5 µL of a plasma sample (calibration samples, QCs or PK samples) was extracted with 10 µL of water containing 10% formic acid, and 15 µl of acetonitrile containing 12.5 ng/mL caffeine as internal standard for 5 min at 2000 rpm on an Eppendorf MixMate® vortex mixer. A 50 µL of a lung sample (calibration samples, QCs or PK samples) was extracted with 15 µL water containing 10% formic acid, and 35 µL of acetonitrile containing 12.5 ng/mL caffeine as internal standard for 5 min at 2000 rpm on an Eppendorf MixMate® vortex mixer. BALF: A 200 µL of methanol were added to 100 µL of a BALF sample (calibration samples, QCs or PK samples). Samples were concentrated in an Eppendorf concentrator until dryness at room temperature. A 90 µL of water containing 10% formic acid and 10 µL of acetonitrile containing 125 ng/mL caffeine were added to the dried samples and dissolved for 20 min at 2,000 rpm on an Eppendorf MixMate® vortex mixer. All samples were centrifuged for 10 min at 4 °C at 13,000 rpm. Supernatants were transferred to standard HPLC-glass vials. HPLC conditions were as follows: column: Agilent Zorbax Eclipse Plus C18, 50x2.1 mm, 1.8 µm; temperature: 30 °C; injection volume: 10 µL; flow rate: 700 µL/min; solvent A: water + 0.1% formic acid; solvent B: acetonitrile + 0.1% formic acid; gradient for **4** and Tob: 99% A at 0 min, 99% – 0% A from 0.1 min to 4.00 min, 0% A until 4.50 min, 0% – 99% A from 4.50 to 4.70 min. Mass spectrometric conditions were as follows: Scan type: MRM, positive mode; Q1 and Q3 masses for caffeine, **4** and Tob can be found in Table S6; peak areas of each sample and of the corresponding internal standard were analyzed using MultiQuant 3.0 software (AB Sciex). Peak areas of the respective sample of **4** and Tob were normalized to the internal standard peak area. Peaks of PK samples were quantified using the calibration curve. The accuracy of the calibration curve was determined using QCs independently prepared on different days (Table S5). PK parameters were determined using a non-compartmental analysis with PKSolver.[9] ELF concentrations were calculated using equations (S3) and (S4).[10]

$$V_{ELF} = V_{BALF} \times \frac{Urea_{BALF}}{Urea_{Plasma}} \, (S3)$$

$$c_{ELF} = c_{BALF} \times \frac{V_{BALF}}{V_{ELF}} \, (S4)$$

Table S5. *Detection limits for LC-MS Analysis of in vivo PK samples.*

ID	limits of quantification	lower limit of	accuracy [%]

	[ng/mL] ([µM])	qualification [ng/mL] ([µM])	
**4**			
plasma	0.5-8000 (0.0012-19.4)	0.25 (0.0006)	94.29-113.66
lung	5-4000 (0.012-9.7)	4 (0.0097)	92.92-109.16
BALF	40-8000 (0.097-19.4)	40 (0.097)	86.76-114.55
Tobramycin			
plasma	40-8000 (0.085-17.1)	25 (0.053)	88.23-114.15
lung	40-8000 (0.085-17.1)	25 (0.053)	89.25-111.42
BALF	40-8000 (0.085-17.1)	25 (0.053)	86.13-113.03

Table S6. *Tuning details for LC-MS Analysis of in vivo PK samples.*

ID	Q1 Mass [Da]	Q3 Mass [Da]	time [msec]	CE [volts]	CXP [volts]	DP [volts]
**4**	411.981	222.100	50	29.0	18.0	130.0
**4**	411.981	163.100	50	31.0	20.0	130.0
Tobramycin	468.164	163.000	30	1.0	29.0	18.0
Tobramycin	468.164	324.000	30	1.0	21.0	36.0
Tobramycin	468.164	205.000	30	1.0	29.0	22.0
Urea	60.915	43.800	30	17.0	16.0	56.0
Urea	60.915	43.100	30	53.0	12.0	56.0
Urea	60.915	29.100	30	111.0	6.0	56.0
Caffeine	195.024	138.000	50	25.0	14.0	130.0
Caffeine	195.024	110.000	50	31.0	18.0	130.0

Table S7. *PK parameter plasma.*

	**4**	Tobramycin
$t_{1/2}$ [h]	0.86 ± 0.4	0.76 ± 0.8
T_{max} [h]	0.5 ± 0.0	0.50 ± 0.0
C_{max} [ng/mL] ([µM])	62.58 ± 45.7 (0.151 ± 0.11)	89.55 ± 25.3 (0.191 ± 0.05)
AUC_{0-t} [ng/mL·h] ([µM]·h)	55.75 ± 21.3 (0.135 ± 0.05)	83.16 ± 35.3 (0.178 ± 0.08)

MRT [h]	1.20 ± 0.1	1.45 ± 1.2
Vz/F_obs [L/kg]	10.46 ± 1.2	2.25 ± 1.6
Cl/F_obs [mL/min/kg]	157.13 ± 55.5	45.31 ± 26.6

Table S8. *PK parameter lung.*

	**4**	Tobramycin
$t_{1/2}$ [h]	1.08 ± 0.5	1.13
T_{max} [h]	0.50 ± 0.0	0.50
C_{max} [ng/g] ([µmol/g])	9856.67 ± 4623.1 (0.024 ± 0.01)	267.56 (0.00057)
AUC_{0-t} [ng/g·h] ([µmol/g·h])	7357.00 ± 2369.4 (0.018 ± 0.005)	230.11 (0.00049)
MRT [h]	1.27 ± 0.4	1.94

Table S9. *PK parameter BALF*

	**4**	Tobramycin
$t_{1/2}$ [h]	0.72 ± 0.2	0.82 ± 0.1
T_{max} [h]	0.50 ± 0.0	0.50 ± 0.0
C_{max} [ng/mL] ([µM])	3770.00 ± 2662.9 (9.2 ± 6.5)	215.43 ± 49.5 (0.46 ± 0.1)
AUC_{0-t} [ng/mL·h] ([µM]·h)	4844.88 ± 2185.2 (11.8 ± 5.3)	267.50 ± 27.3 (0.57 ± 0.06)
MRT [h]	1.57 ± 0.1	1.40 ± 0.1

Table S10. *PK parameter ELF*

	**4**	Tobramycin
$t_{1/2}$ [h]	0.55 ± 0.3	0.56 ± 0.2
T_{max} [h]	1.67 ± 2.0	0.50 ± 0.0
C_{max} [ng/mL] ([µM])	1286.91 ± 298.7 (3.1 ± 0.7)	38.77 ± 8.3 (0.083 ± 0.02)
AUC_{0-t} [ng/mL·h] ([µM]·h)	3095.83 ± 330.2 (7.5 ± 0.8)	57.54 ± 14.7 (0.12 ± 0.03)
MRT [h]	1.61 ± 1.1	1.50 ± 0.2

6.3. Design of *in vivo* Target Engagement Study

General. Mouse experiments were approved by the Landesamt für Soziales, Gesundheit und Verbraucherschutz of the State of Saarland in accordance with the national guidelines for animal treatment on August, 17th, 2017 (22/2017). The IVC unit was located within a biosafety level 2 laboratory and mice were allowed to acclimatize to their environment for at least seven days prior to the experiment. Throughout the experiment, the mice had access to water and food ad libitum.

Background studies were performed in order to generate typical mean and standard deviation values for biomarkers of the control group. Sample size was calculated using BiAS. (Software Version 11.08) from these values with alpha = 0.05 and P = 0.8 for biomarker reduction of 80% resulting in a sample size of ten mice or higher for a two-sample t-test.

Primary study. Ten-week old female C57BL/6N mice were housed in an individually ventilated-cage unit with ten mice per cage and twenty mice in total. One group (from one cage) was treated with compound **4**, while the other group received a sham treatment with vehicle. Mice were not randomized, and no blinding was applied.

For the infection, each mouse was anesthetized intraperitoneally with 2.025 mg of ketaminhydrochloride (Ursotamin, Serumwerk Bernburg, Germany) and 0.12 mg of xylazinhydrochloride (Xylazin, WDF, Serumwerk Bernburg). Lidocainhydrochloride (Xylocain; Aspen Pharma, Ireland) was used for local anesthesia of the epiglottis before endotracheal intubation with a flexible cannula and intra-tracheal application of the inoculum and/or treatment.

Application of inoculum (start of infection, 0 h) using 20 µL viable *P. aeruginosa* NH57388A culture supplemented with sodium alginate (22 mg/mL, Sigma). These were mixed with 20 µL of either compound **4** (5 mg/kg BW suspended in 0.1% methylcellulose) or vehicle (0.1% methylcellulose) to conduct the first treatment simultaneously to infection via intra-tracheal application assisted by endotracheal intubation using a flexible cannula. Bacterial load was determined to be $5{\cdot}10^7$ CFU per mouse by plating serial dilutions on LB-agar plates. Subsequent treatments were performed at 24 and 48 h post infection with 20 µL of compound **4** (5 mg/kg BW suspended in 0.5% methylcellulose, 0.2 vol-% Tween-80) or vehicle (0.5% methylcellulose, 0.2 vol-% Tween 80) were administered intra-tracheally following the same procedure as the infection. 72 h post infection mice were euthanized, lungs were removed aseptically and homogenized in 500 µL sterile phosphate buffered saline over the course of

72 h. Again, bacterial load was determined by plating serial dilutions on LB-agar plates. The determined mean CFU values are given below (Table S11). Survival rate of mice was 100% in the vehicle group and 70% in the compound **4** treatment group, however, 30% of animals were lost in the vehicle group due to technical problems with the intubation. Initial body weight was 19.9 ± 0.9 g and weight loss was 16.1% at 48 h post infection and 23.6% immediately before euthanization. Animal appearance was within the predefined animal health score.

Quantification of Alkylquinolones from lung homogenates. Alkylquinolone (AQ) signal molecules in lung homogenates were analyzed after ethyl acetate extraction *via* the LC-ESI-MS/MS method described above.

Table S11. *Alkylquinolone and CFU data from in vivo target engagement experiment.*

Groups	HHQ (pmol)	HQNO (pmol)	PQS (pmol)	CFU/mouse
	1.0	2.4	0*	33300
	0.2*	0.7*	0.2*	31400
	0.9	2.3	0.1*	147500
Treated	0.1*	0*	0.1*	436700
	1.0	2.3	0.2*	207100
	8.6	27.0	1.3*	72500
	0.5	1.5	0.3*	286700
Mean	1.7	5.2	0.3*	173600
SD	3.1	9.7	0.5	149412
Median	0.9	2.3	0.2*	147500
	615.7	835.0	91.3	105000
	223.5	247.9	23.9	143300
	189.7	182.3	23.2	281430
Untreated	3.2	6.9	0.4	221430
	677.6	783.4	96.3	1500000
	7.5	10.9	1.5	1307000
	23.4	39.2	2.4	1800000
Mean	248.6	300.8	34.1	765451
SD	286.4	359.2	42.0	736731
Median	189.7	182.3	23.2	281430
p-value F-Test	0.000	0.000	0.000	0.001
p-value t-Test	0.031	0.036	0.038	0.039
Reduction through treatment (%)	99.3	98.3	99.1	77.3
Pearson‘s r HHQ vs. HQNO	0.9943			
Pearson‘s r HHQ vs. PQS	0.9971			
Pearson‘s r PQS vs. HQNO	0.9968			

*below lower limit of quantification (LLOQ): ~0.25 pmol HHQ and HQNO, ~2.5 pmol PQS

7. Xray Crystallography

7.1. Expression and Purification of $PqsR^{91\text{-}319}$

The plasmid pOPIN-His_6-SUMO-$PqsR^{91\text{-}319}$ was transformed into *E. coli* BL21-CodonPlus(DE3)-RIL, and protein expression in TB (+Amp+Cm) was induced with 0.5 mM IPTG for 16 h at 20 °C, when the culture reached an OD_{600} of 0.6–0.8. After centrifugation, the cells were resuspended in buffer A (20 mM HEPES 8.0, 300 mM NaCl, 5mM BME) supplemented with one EDTA-free protease inhibitor cocktail tablet (Roche Life Science). The cells were lysed with an Emulsiflex-C3 homogenizer (Avestin) with two cycles. Supernatant was separated from pellet by centrifugation in a SA-60 rotor at 16,000 rpm for 30 min. The supernatant was applied onto a 5 ml HisTrap HP column (GE Healthcare). His_6-SUMO-$PqsR^{91\text{-}319}$ was eluted with a linear gradient (20 mM HEPES pH 8.0, 300 mM NaCl, 0-250 mM imidazole pH 8.0). His_6-SUMO-$PqsR^{91\text{-}319}$ containing fractions were pooled and after cleavage o.n. of the His_6-SUMO tag by SUMO protease (1%, *e.g.*, 0.01 mg SUMO protease per 1 mg of His_6-SUMO-$PqsR^{91\text{-}319}$; dialyse in parallel against buffer without imidazole) and a second (reverse) nickel affinity chromatography, $PqsR^{91\text{-}319}$ was finally purified by size exclusion chromatography in 20 mM HEPES, 150 mM NaCl, 1 mM DTT, pH 8.0. The protein was concentrated and flash-frozen in liquid nitrogen and stored at -80 °C.

7.2. Protein Crystallization, Data Collection and Structure Solution

Crystal of $PqsR^{91\text{-}319}$ in complex with compound **4** were obtained by co-crystallization in a sitting drop vapor diffusion set-up. Prior crystallization 10 mg/ml $PqsR^{91\text{-}319}$ was mixed with 1.5 mM compound **4** and 0.4 mM of Ponceau 4 R and incubated for 10 min prior centrifugation at 14,000 rpm for 5 min. Equal amounts of protein were mixed with 0.15 M $MgCl_2$, 1.18 M LiCl and 0.1 M MES pH 6.2. Crystals grew to full size (120x50x50 µm) within 24 days at 20 °C. The crystals were cryoprotected with 20% (v/v) 2,3-Butanediol and flash-frozen in liquid nitrogen. X-ray diffraction data were collected at 100 K at P11 of PETRA III synchrotron (Table S12). Diffraction data were processed with autoPROC pipeline, including *XDS,*[11] *AIMLESS,*[12] *and STARANISO.*[13] The data was corrected for anisotropy using the STARANISO output from autoPROC. The structures were determined by molecular replacement with *PHASER*[14] using the PDB entry 6Q7V. Model building and

refinement were performed with *COOT*[15] and with *phenix.refine*[16] from the *PHENIX* suite,[17] respectively.

Table S12. Data collection and refinement statistics.

	$PqsR^{91\text{-}319}$ – **4***
**Data collection statistics**	
Beamline[a]	P11, PETRA III
Wavelength (Å)	1.0332
Space group	$C222_1$
Unit cell dimensions	
a, b, c (Å)	109.8, 120.6, 113.2
α, β, γ (°)	90.0, 90.0, 90.0
Resolution range (Å)	60.32-2.16 (2.45-2.16)
Ellipsoidal[a] resolution (Å) (direction)[b]	3.00 (a*)<br>3.00 (b*)<br>2.09 (c*)
Mosaicity (°)	0.20
Total No. of	
measured reflections	267132 (10868)** (ellipsoidal)
unique reflections	20478 (1025) (ellipsoidal)
Mean I/σ(I)	15.0 (1.6) (ellipsoidal)
Multiplicity	13.0 (10.6)
Completeness (spherical) (%)	50.6 (8.1)
Completeness (ellipsoidal) (%)	93.6 (73.9)
R_{meas}[c] (%)	11.8 (160.0)
$R_{p.i.m.}$[d] (%)	3.2 (48.5)
$CC_{1/2}$[e]	99.9 (54.0)
Solvent content (%)	66.5
Monomers/ASU	2
**Refinement statistics**	
Resolution range (Å)	60.32-2.16 (2.28-2.16)
R_{work} (%)	20.3
R_{free} (%)	26.0
No. of non H-atoms	
Protein	3307
MgO_6	7
Compound 4	60
Ponceau 4 R	70
Water	64
R.m.s. deviations	
Bonds (Å)	0.014
Angles (°)	1.593
Average B factors (Å^2)	

Protein	47.6
MgO_6	32.9
Compound 4	36.1
Ponceau 4 R	36.2
Water	36.9
Ramachandran plot (%)	
Favored regions	93.2
Outliers	0.24
MolProbity score	2.45
PDB ID	6YIZ

*Data for PqsR$^{91\text{-}319}$ in complex with **4** were processed anisotropically with STARANISO via the autoPROC[18] pipeline.

**Values in parentheses refer to the highest resolution shell.

[a] The statistics are for data that were corrected for anisotropy by STARANISO.[13]

[b] The resolution limits for three directions in reciprocal space (a*, b*, c*) are indicated here. To accomplish this, STARANISO[1] computed an ellipsoid postfitted by least squares to the cutoff surface, removing points where the fit was poor. Note that the cutoff surface is unlikely to be perfectly ellipsoidal, so this is only an estimate.

[c] $R_{meas} = \Sigma_{hkl} [N / (N - 1)]^{1/2} \Sigma_i |I_i(hkl) - \langle I(hkl)\rangle| / \Sigma_{hkl} \Sigma_i I_i(hkl)$, where N is the multiplicity, $I_i(hkl)$ is the intensity of the ith measurement of the reflection hkl and $\langle I(hkl)\rangle$ is the mean intensity of multiple observations of the reflection hkl.

[d] $R_{p.i.m.} = \Sigma_{hkl} [1 / (N - 1)]^{1/2} \Sigma_i |I_i(hkl) - \langle I(hkl)\rangle| / \Sigma_{hkl} \Sigma_i I_i(hkl)$.

[e] $CC_{1/2}$: Correlation coefficient between the intensities of two random half data sets.

8. Analysis and Visualization of complex structures of compounds 2 and 4 bound to PqsR$^{91\text{-}319}$

All 3D structures were prepared with MOE 2019.0102 (Molecular Operating Environment, Chemical Computing Group),[19] while graphic processing for manuscript figures was done using YASARA structure (YASARA Biosciences GmBH)[20] and POV-Ray 3.7.0.

For **Figure 1C**, 6Q7W was loaded into MOE, and the amide motif of compound (**2**) was modelled into the ligand structure using the built-in “Builder” function of MOE followed by a refinement step using the built-in “QuickPrep” function with standard parameters and the Amber10:EHT force field.

For **Figure 2A-B**, newly solved complex structure (PDB ID: 6YIZ) was loaded into MOE followed by a refinement step using the built-in “QuickPrep” function with standard parameters and the Amber10:EHT force field. 2D interaction profile was generated using the built-in “Ligand Interactions” function of MOE.

9. Statistics

Statistical Analysis

Results of biological experiments are reported as means of at least three independent experiments, unless otherwise stated.

For MBEC determination, One-way ANOVA was performed using GraphPad Prism 8.4.2.

For animal studies, unpaired t-test (one-sided, p-value) was performed with Microsoft Excel.

A summary of data processing and statistical analysis is shown in Table S13. Detailed information can be found in the respective experimental section.

The CEREP panel, determination of CYP and hERG inhibition as well as Mouse MTD were performed at CROs.

Table S13. Details on the analysis for different assays used.

Test	Preprocessing	Presentation*	N	Statistical Method	Software
Cytotoxicity Assays	Normalization to DMSO control, mean of 2 technical replicates	Mean % viable cells	2	-	Microsoft Excel
Kinetic Turbidimetric Solubility	Blank subtraction	Mean	3	AUC Determination	GraphPad Prism 8.4.2
Metabolic Stability	Mean of 2 technical replicates	Mean ± SD	2	Linear regression of semi-logarithmic plot	Microsoft Excel
Permeability	mass correction for cumulative sampling	Mean P_{app} ± standard deviation	N = 3 individual experiments with each 3-4 replicates	none	Excel
Nanoparticle Preperation	Minimum of three independent experiments with technical triplicates	Mean ± SD	3	-	Zetasizer software v7.02, Malvern Instruments Ltd.
Release study	Three independent experiments with technical triplicates	Mean ± SD	3	-	Microsoft Excel
MBEC	Four independent experiments with four technical replicates (n=4)	Scatter plot with mean and SEM	4	One-way ANOVA, / AUC determination	OriginPro 2020

Pyocyanin assay	Blank subtraction, Normalization to cell growth	Dose response curve, mean ± SD/CI	3	Non-linear regression	Microsoft Excel, OriginPro 2020
Pharmacokinetics	Normalization of peak area to internal standard peak area, calculation of conc. based on calibration curve	Scatter plot including mean concentration vs. time; mean ± SE	3	-	Microsoft Excel PK Solver
Alkylquinolone Inhibition Assay	Normalization of peak area to internal standard, mean of 3 technical replicates, calculation of concentration based on a calibration curve	Dose response curve, mean w/o error	3	Non-linear regression	Microsoft Excel, OriginPro 2020
In vivo Target Engagement (*in vivo* AQ levels)	Concentration calculated from peak area then transformed to absolute amounts per organ via organ weight	Single values S11, Figure 3D	7 + 7	Unpaired t-test, one-sided, p-value	Microsoft Excel, BiAS. for group size determination
In vivo CFU	Normalization to volume used for counting, normalized per animal	Single values Suppl. Table 11	7 + 7 (treated vs. control)	Unpaired t-test, two-tailed p-value	GraphPad Prism 8.4.2
Reporter Gene Assay	Normalization to solvent control	Mean IC_{50}/CI	≥2	Non-linear regression	OriginPro 2020
X-ray	See Table S12				

*SD = Standard deviation; CI = confidence interval of 95%

10. References

[1] K. Barral, A. D. Moorhouse, J. E. Moses, *Organic letters* **2007**, *9*, 1809.

[2] D.-K. Ho, X. Murgia, C. de Rossi, R. Christmann, A. G. Hüfner de Mello Martins, M. Koch, A. Andreas, J. Herrmann, R. Müller, M. Empting et al., *Angewandte Chemie (International ed. in English)* **2020**, *59*, 10292.

[3] C. Lu, B. Kirsch, C. Zimmer, J. C. de Jong, C. Henn, C. K. Maurer, M. Müsken, S. Häussler, A. Steinbach, R. W. Hartmann, *Chemistry & biology* **2012**, *19*, 381.

[4] C. Cugini, M. W. Calfee, J. M. Farrow, D. K. Morales, E. C. Pesci, D. A. Hogan, *Molecular microbiology* **2007**, *65*, 896.

[5] M. P. Storz, C. K. Maurer, C. Zimmer, N. Wagner, C. Brengel, J. C. de Jong, S. Lucas, M. Müsken, S. Häussler, A. Steinbach et al., *Journal of the American Chemical Society* **2012**, *134*, 16143.

[6] M. Zender, F. Witzgall, A. Kiefer, B. Kirsch, C. K. Maurer, A. M. Kany, N. Xu, S. Schmelz, C. Börger, W. Blankenfeldt et al., *ChemMedChem* **2019**.

[7] J. J. Harrison, C. A. Stremick, R. J. Turner, N. D. Allan, M. E. Olson, H. Ceri, *Nature protocols* **2010**, *5*, 1236.

[8] J. Haupenthal, C. Baehr, S. Zeuzem, A. Piiper, *International journal of cancer* **2007**, *121*, 206.

[9] Y. Zhang, M. Huo, J. Zhou, S. Xie, *Computer methods and programs in biomedicine* **2010**, *99*, 306.

[10] S. Kiem, J. J. Schentag, *Antimicrobial agents and chemotherapy* **2008**, *52*, 24.

[11] W. Kabsch, *Acta crystallographica. Section D, Biological crystallography* **2010**, *66*, 125.

[12] P. R. Evans, G. N. Murshudov, *Acta crystallographica. Section D, Biological crystallography* **2013**, *69*, 1204.

[13] Tickle, I.J., Flensburg, C., Keller, P., Paciorek, W., Sharff, A., *STARANISO*, Cambridge, United Kingdom: Global Phasing Ltd, **2018**.

[14] A. J. McCoy, R. W. Grosse-Kunstleve, P. D. Adams, M. D. Winn, L. C. Storoni, R. J. Read, *Journal of applied crystallography* **2007**, *40*, 658.

[15] P. Emsley, B. Lohkamp, W. G. Scott, K. Cowtan, *Acta crystallographica. Section D, Biological crystallography* **2010**, *66*, 486.

[16] P. V. Afonine, R. W. Grosse-Kunstleve, N. Echols, J. J. Headd, N. W. Moriarty, M. Mustyakimov, T. C. Terwilliger, A. Urzhumtsev, P. H. Zwart, P. D. Adams, *Acta crystallographica. Section D, Biological crystallography* **2012**, *68*, 352.

[17] P. D. Adams, P. V. Afonine, G. Bunkóczi, V. B. Chen, I. W. Davis, N. Echols, J. J. Headd, L.-W. Hung, G. J. Kapral, R. W. Grosse-Kunstleve et al., *Acta crystallographica. Section D, Biological crystallography* **2010**, *66*, 213.

[18] C. Vonrhein, C. Flensburg, P. Keller, A. Sharff, O. Smart, W. Paciorek, T. Womack, G. Bricogne, *Acta crystallographica. Section D, Biological crystallography* **2011**, *67*, 293.

[19] *Molecular Operating Environment (MOE)*, Chemical Computing Group ULC, **2013**.

[20] E. Krieger, G. Koraimann, G. Vriend, *Proteins* **2002**, *47*, 393.
